# Advancements in the mechanistic understanding of the copper-catalyzed azide–alkyne cycloaddition

**DOI:** 10.3762/bjoc.9.308

**Published:** 2013-12-02

**Authors:** Regina Berg, Bernd F Straub

**Affiliations:** 1 Organisch-Chemisches Institut, Ruprecht-Karls-Universität Heidelberg, Im Neuenheimer Feld 270, 69120 Heidelberg, Germany

**Keywords:** alkyne, azide, Click, copper, CuAAC, DFT study, Huisgen–Meldal–Sharpless cycloaddition, kinetics, reaction mechanism

## Abstract

The copper-catalyzed azide–alkyne cycloaddition (CuAAC) is one of the most broadly applicable and easy-to-handle reactions in the arsenal of organic chemistry. However, the mechanistic understanding of this reaction has lagged behind the plethora of its applications for a long time. As reagent mixtures of copper salts and additives are commonly used in CuAAC reactions, the structure of the catalytically active species itself has remained subject to speculation, which can be attributed to the multifaceted aggregation chemistry of copper(I) alkyne and acetylide complexes. Following an introductory section on common catalyst systems in CuAAC reactions, this review will highlight experimental and computational studies from early proposals to very recent and more sophisticated investigations, which deliver more detailed insights into the CuAAC’s catalytic cycle and the species involved. As diverging mechanistic views are presented in articles, books and online resources, we intend to present the research efforts in this field during the past decade and finally give an up-to-date picture of the currently accepted dinuclear mechanism of CuAAC. Additionally, we hope to inspire research efforts on the development of molecularly defined copper(I) catalysts with defined structural characteristics, whose main advantage in contrast to the regularly used precatalyst reagent mixtures is twofold: on the one hand, the characteristics of molecularly defined, well soluble catalysts can be tuned according to the particular requirements of the experiment; on the other hand, the understanding of the CuAAC reaction mechanism can be further advanced by kinetic studies and the isolation and characterization of key intermediates.

## Introduction

**Scheme 1:**
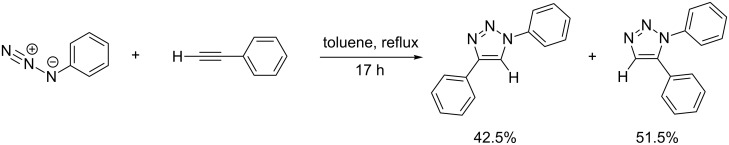
Exemplary 1,3-dipolar cycloaddition of phenylacetylene with phenyl azide [6].

In 1893, Michael discovered a reaction between dimethyl but-2-ynedioate and phenyl azide at 100 °C in a sealed tube and suggested that regioisomeric triazoles were formed [1]. However, it was only in the 1960s that Huisgen recognized this type of reaction for its generality, scope and mechanism [2–5], and coined the term 1,3-dipolar cycloaddition. The classical thermal Huisgen cycloaddition of organoazides and alkynes proceeds very slowly even at high temperatures, and gives a mixture of 1,4- and 1,5-disubstituted 1,2,3-triazoles (Scheme 1).

In 2002, the groups of Meldal and Sharpless independently discovered a copper-catalyzed variant of Huisgen’s azide–alkyne cycloaddition (CuAAC reaction). In fact, the catalytic effect of copper ions had first been mentioned by L’Abbé in 1984 [7], but had henceforth been overlooked until Meldal presented a copper(I)-catalyzed solid-phase synthesis of 1,2,3-triazoles. In this procedure, the terminal alkyne substrate is bound to a hydrophilic tertiary amide-poly(ethylene glycol) based resin via a peptide linker [8]. With copper(I) salts as catalysts, the corresponding triazole is formed under mild conditions upon addition of the azide. This reaction proceeds in a variety of organic solvents at room temperature with quantitative conversion to give the 1,4-disubstituted 1,2,3-triazole exclusively. Common side reactions such as the Glaser coupling [9–11] are not observed. The presented reaction conditions are compatible with a variety of functional groups such as ester, ether, amide, thioether, Fmoc and Boc groups. Meldal et al. reported this reaction in the context of solid-supported peptide synthesis and expressed their hope for the preparation of a library with triazole-containing peptides.

**Scheme 2:**
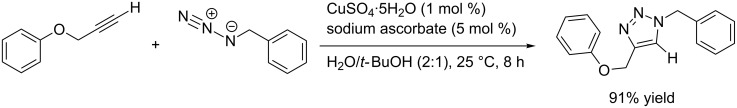
CuAAC reaction of benzyl azide with (prop-2-yn-1-yloxy)benzene [12].

The group of Sharpless, on the other hand, presented a copper-catalyzed azide–alkyne cycloaddition under solution-phase conditions (Scheme 2) [12]. In their standard procedure, the cost-efficient salt copper(II) sulfate pentahydrate is reduced in situ by ascorbic acid or sodium ascorbate in a solvent mixture of water and alcohol (“Sharpless–Fokin conditions”). Alternatively, copper(I) salts such as copper(I) iodide, copper(I) triflate or tetrakis(acetonitrile)copper(I) hexafluorophosphate can be used in the presence of a nitrogen base with acetonitrile as co-solvent. This reaction is applicable to a great variety of substrates with manifold functional groups, e.g. free hydroxy, ester, carboxylic acid, amide, sulfonamide and amine substituents. The catalytic process is insensitive towards the presence of air and pH changes between pH 4 and 12 in a solvent mixture of water and *tert*-butanol. This strictly regioselective stepwise process gives the 1,4-disubstituted 1,2,3-triazole only and accelerates the reaction by a factor of up to 10^7^ in comparison to Huisgen’s uncatalyzed procedure [13–14].

Since Meldal and Sharpless had first reported this copper-catalyzed variant of Huisgen’s 1,3-dipolar cycloaddition, a myriad of protocols employing different catalyst systems has been described. It is essential for the discussion of the reaction’s mechanistic details to introduce some of the catalytically active reagent mixtures used in CuAAC, as the choice of reagents and especially the presence of ligands [15–16] does strongly influence speciation, nuclearity and solubility of the copper(I) species involved in the catalytic cycle.

## Review

We will summarize the development of the mechanistic understanding of the copper-catalyzed azide–alkyne cycloaddition from early proposals to a more sophisticated updated view based on results from kinetic and computational studies in the last decade. Some sections of this review are also part of the dissertation of one of the authors [17].

The use of ligands in copper-catalyzed [3 + 2] azide–alkyne cycloadditions has recently been excellently reviewed [15–16]. Nonetheless, we will give a short outline of the reagent mixtures and copper(I) complexes commonly employed in order to understand the problems as to speciation and nuclearity faced by mechanistic studies. More general aspects of the CuAAC as well as the multitude of fields, in which this reaction has become essential, are highlighted in a great many of recommendable review articles [14,18–42] and will not be subject of this article.

### Common reagents for CuAAC catalysis

Common CuAAC precatalyst mixtures contain elemental copper, copper(II) salts or copper(I) species. In all cases, however, the catalytically active species contains copper in the oxidation state +I [8,12,19].

#### CuAAC catalysis with copper(0) precatalysts

The CuAAC reaction proceeds in the presence of coiled copper metal turnings at room temperature by in situ oxidation to copper(I) species [12–13], even though this route takes longer for completion (12–48 hours) than with the standard catalyst systems, i.e. copper(II) salts plus reducing agent or copper(I) salts [14]. This method can be significantly sped up by applying microwave radiation [43]. It is also beneficial to add copper(II) sulfate, but this is usually not mandatory as the patina on the copper surface is sufficient to start the catalysis [14].

The group of Rothenberg introduced nanometric copper clusters for ligand-free CuAAC [44]. These copper nanoclusters are prepared by reduction of cuprous chloride. Compared to other catalytic systems such as copper powder, copper shavings or copper(II) sulfate/ascorbate, the reaction with nanocluster catalysts proceeds faster, which is probably due to their higher surface area that favours heterogeneous catalysis.

The main advantage of using copper metal or nanoclusters is the high purity of the products, as the latter do only contain minute remainders of copper.

#### CuAAC catalysis with mixtures of copper(II) salts and additives

The most common source of copper for the CuAAC reaction are copper(II) salts. In the standard procedures introduced by Sharpless and Fokin [12], copper(II) sulfate pentahydrate is reduced in situ by sodium ascorbate, which is added in three- to ten-fold excess (Scheme 2) [18]. This procedure is tolerant towards most functional groups, even free amines, alcohols and carboxylic acids, and can be carried out in aqueous reaction media with organic co-solvents such as alcohols or dimethyl sulfoxide. As an alternative reducing agent, the water-soluble tris(2-carboxyethyl)phosphine (TCEP; often prepared and used as its hydrochloride salt TCEP·HCl) can be employed [45–55], which is especially suitable for applications in biological systems because it also protects cysteine residues in proteins from oxidative coupling [45–46]. However, the latter phosphine reagent should only be used in low quantities, as its binding to copper ions has an inhibitory effect. Another disadvantage of using TCEP is the consumption of the azide substrates in Staudinger reactions [56–58] with the phosphine, giving the corresponding amine and tris(2-carboxyethyl)phosphine oxide as byproducts [59–61].

The main advantages of reducing copper(II) salts in situ are the broad applicability of this procedure and its compatibility with oxygen and water, which means that there is no need for inert gas conditions. Together with the usually very high yields, the lack of byproducts and uncomplicated work-up procedures, CuAAC reactions with copper(II) salts reduced in situ fulfil all criteria in the concept of “Click” chemistry [62].

However, application of these protocols is naturally limited to substrates stable towards aqueous conditions, and the procedures can hardly be modulated according to specific requirements. As for mechanistic investigations, the nature of the catalytically active copper(I) complex is unknown and there is scarcely any chance to rationally explain the results of kinetic studies.

In order to protect the copper(I) ions from disproportionation to Cu(0) and Cu(II) and from re-oxidation to Cu(II) by air, to enhance their catalytic activity and to improve the reaction’s applicability with a variety of substrates, the search for suitable ligands started immediately after Sharpless’ and Meldal’s initial reports [8,12].

**Scheme 3:**
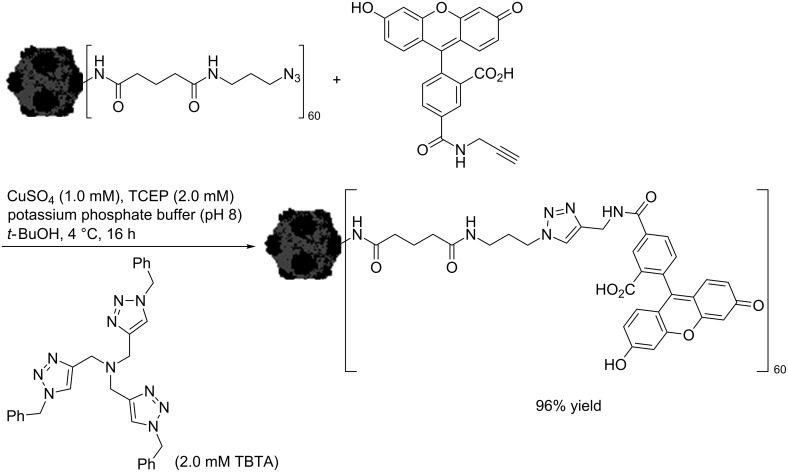
Bioconjugation reaction of capsid-bound azide groups with alkynyl-functionalized dye molecules (cowpea mosaic virus is presented by an image of its crystal structure as published by the group of Lin in 1999) [45,67].

Shortly after their seminal communication on the CuAAC reaction, the group of Sharpless reported the observation of an autocatalytic effect in the synthesis of tris(triazolylmethyl)amines, i.e. the tris(triazolylmethyl)amine products act as rate-accelerating ligands [45]. The authors used the newly developed ligand system in a bioconjugation reaction with a virus. The exterior surface of the coat protein of cowpea mosaic virus was labelled with 60 azide groups as shown in Scheme 3 [45]. Then, the reaction with an alkyne-functionalized dye was carried out in the presence of copper(II) sulfate, tris[(1-benzyl-1*H*-1,2,3-triazol-4-yl)methyl]amine (TBTA) [63] as ligand and TCEP as reducing agent in a solution of potassium phosphate buffer (pH 8) and *tert*-butanol at 4 °C. Apart from accelerating the CuAAC process, the key function of TBTA is to stabilize the Cu(I) oxidation state in aqueous solution [14,64]. This is important as Cu(II) ions are harmful to this experiment in two ways: on the one hand, Cu(II) ions catalyze the oxidative coupling of the alkyne substrates to give diynes as undesired byproducts (Glaser coupling [9–11], Eglinton coupling [11,65–66]); on the other hand, Cu(II)-triazole adducts on the protein surface of the virus induce the rapid decomposition of the capsid [45].

This communication by Sharpless et al. in 2003 triggered the search for other highly effective polytriazole ligands in combination with copper(II) salts.

In 2004, Sharpless and Fokin compared a variety of polytriazoles as ligands in the CuAAC model reaction of benzyl azide with phenylacetylene in a solvent mixture of water and *tert*-butanol under aerobic conditions [64]. However, of all candidates tested, TBTA turned out to be the most potent additive for protecting the copper(I) ions with regard to oxidation and disproportionation. It was supposed that TBTA acts as a tetradentate ligand and blocks all coordination sites at the metal centre so that no oxidant can attack at the copper(I) ion. The tertiary amine’s nitrogen atom was assumed to bind permanently to the metal centre, while the pendant triazole ligands might temporarily dissociate from the copper(I) ion to allow for the formation of the copper acetylide as starting point of the catalytic cycle.

**Figure 1:**
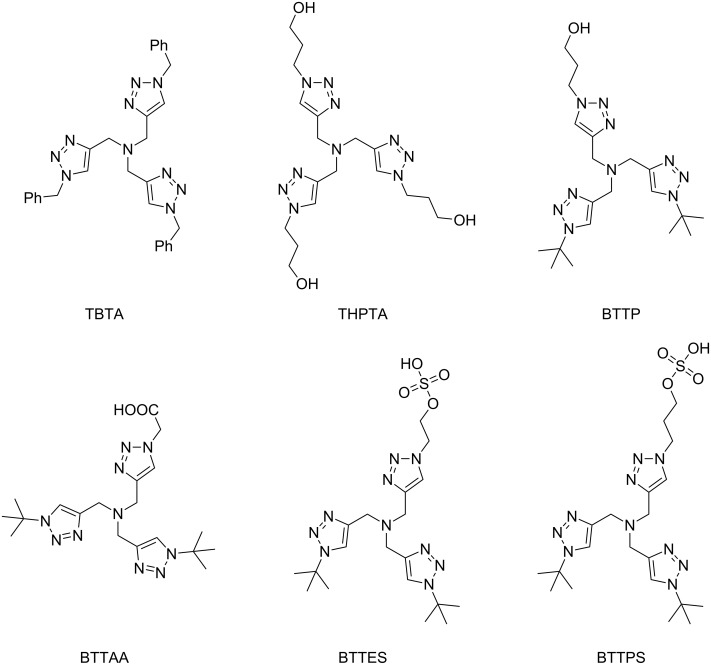
Tris(triazolylmethyl)amine ligands for CuAAC applications in bioorganic chemistry: TBTA = tris[(1-benzyl-1*H*-1,2,3-triazol-4-yl)methyl]amine [45,63–64]; THPTA = tris[(1-hydroxypropyl-1*H*-1,2,3-triazol-4-yl)methyl]amine [70–78]; BTTP = 3-[4-{(bis[(1-*tert*-butyl-1*H*-1,2,3-triazol-4-yl)methyl]amino)methyl}-1*H*-1,2,3-triazol-1-yl]propanol [79]; BTTAA = 2-[4-{(bis[(1-*tert*-butyl-1*H*-1,2,3-triazol-4-yl)methyl]amino)methyl}-1*H*-1,2,3-triazol-1-yl]acetic acid [74]; BTTES = 2-[4-{(bis[(1-*tert*-butyl-1*H*-1,2,3-triazol-4-yl)methyl]amino)methyl}-1*H*-1,2,3-triazol-1-yl]ethyl hydrogen sulfate [68]; BTTPS = 3-[4-{(bis[(1-*tert*-butyl-1*H*-1,2,3-triazol-4-yl)methyl]amino)methyl}-1*H*-1,2,3-triazol-1-yl]propyl hydrogen sulfate [79].

The CuAAC reaction can even be carried out with tetrakis(acetonitrile)copper(I) hexafluorophosphate (1 mol %) and TBTA (1 mol %) under aerobic conditions [64]. Cyclic voltammetry measurements have supported the hypothesis of TBTA influencing the redox activity of copper(I), as the redox potential of the Cu(I)/Cu(II) pair was shown to rise by approximately 300 mV when the water-soluble derivative tris(hydroxypropyltriazolylmethyl)amine (THPTA, Figure 1) was present [64].

In bioorganic chemistry, research efforts have focused on developing ligands that can prevent the copper ions from causing biological damage [68–70]. Apart from TBTA that had been shown to increase the biocompatibility of CuAAC in the bioconjugation reaction with cowpea mosaic virus (Scheme 3) [45], water-soluble derivatives such as THPTA [70–78], BTTP [79], BTTAA [74], BTTES [68], and BTTPS [79] have been applied in CuAAC reactions (Figure 1).

Closely related to tris(triazolylmethyl)amines (Figure 1) are different tris(heteroarylmethyl)amine ligands such as tris(pyridylmethyl)amines, tris(benzothiazolylmethyl)amines and tris(2-benzimidazolylmethyl)amines as well as hybrids of the latter (Table 1) [80–83]. These ligands have the same structural motif of a central tertiary amine being surrounded by three nitrogen heterocycles, but provide for CuAAC reactions that are much faster than with TBTA [80].

**Table 1:** Structural variety of tris(heteroarylmethyl)amine ligands synthesized by the group of Finn (the same abbreviations as in the original publication are used: BimY = benzimidazolylmethyl with substituent Y at the N atom; Bth = benzothiazolylmethyl; E = CO_2_Et; E’ = CO_2_*t*-Bu; A = CO_2_^−^K^+^) [80,82].

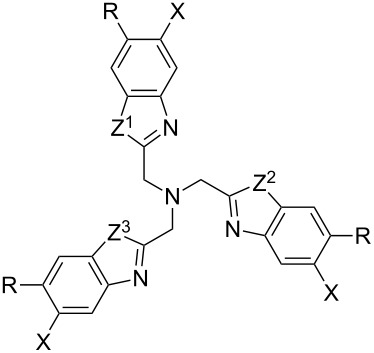					

	Z^1^	Z^2^	Z^3^	X	R

(BimH)_3_	NH	NH	NH	H	H
(Bth)(BimH)_2_	S	NH	NH	H	H
(Bth)_2_(BimH)	S	S	NH	H	H
(BimH/S)_3_	NH	NH	NH	SO_3_H	H
(BimH/Me_2_)_3_	NH	NH	NH	CH_3_	CH_3_
(BimC_1_H)_3_	NCH_3_	NCH_3_	NCH_3_	H	H
(BimC_3_H)_3_	N(CH_2_)_2_Me	N(CH_2_)_2_Me	N(CH_2_)_2_Me	H	H
(BimC_1_E)_3_	N(CH_2_)CO_2_Et	N(CH_2_)CO_2_Et	N(CH_2_)CO_2_Et	H	H
(BimC_1_E’)_3_	N(CH_2_)CO_2_*t*-Bu	N(CH_2_)CO_2_*t*-Bu	N(CH_2_)CO_2_*t*-Bu	H	H
(BimC_1_A)_3_	N(CH_2_)CO_2_K	N(CH_2_)CO_2_K	N(CH_2_)CO_2_K	H	H
(BimC_3_A)_3_	N(CH_2_)_3_CO_2_K	N(CH_2_)_3_CO_2_K	N(CH_2_)_3_CO_2_K	H	H
(BimH)_2_(BimC_4_A)	NH	NH	N(CH_2_)_4_CO_2_K	H	H
(Bth)(BimC_4_A)_2_	S	N(CH_2_)_4_CO_2_K	N(CH_2_)_4_CO_2_K	H	H
(BimC_4_E)_3_	N(CH_2_)_4_CO_2_Et	N(CH_2_)_4_CO_2_Et	N(CH_2_)_4_CO_2_Et	H	H
(BimC_4_A)_3_	N(CH_2_)_4_CO_2_K	N(CH_2_)_4_CO_2_K	N(CH_2_)_4_CO_2_K	H	H
(BimC_4_A/Me_2_)_3_	N(CH_2_)_4_CO_2_K	N(CH_2_)_4_CO_2_K	N(CH_2_)_4_CO_2_K	CH_3_	CH_3_
(BimH)(BimC_5_A)_2_	NH	N(CH_2_)_5_CO_2_K	N(CH_2_)_5_CO_2_K	H	H
(BimC_5_A)_3_	N(CH_2_)_5_CO_2_K	N(CH_2_)_5_CO_2_K	N(CH_2_)_5_CO_2_K	H	H
(Bth)_3_	S	S	S	H	H

As neither copper(I) alkyne π-complexes nor acetylide complexes with this class of tris(heteroarylmethyl)amines as ancillary ligands have been characterized, mechanistic investigations have focused on kinetic measurements. The catalytic activity in the presence of the ligands presented in Table 1 (0.2 mM) was assessed in the test reaction of phenylacetylene (2 mM) with benzyl azide (1 mM) in a solvent mixture of dimethyl sulfoxide and aqueous buffer in the presence of sodium ascorbate (45 mM) and copper(II) sulfate (0.1 mM) at room temperature [80]. Generally, all tris(2-benzimidazolylmethyl)amine ligands greatly accelerate this CuAAC reaction, but the rate is dependent on the nature of the heterocycle as well as on its substitution. As long as at least one benzimidazole ring is present in the ligand, the others can be replaced by benzothiazole rings. The rate of reaction in the CuAAC test reaction increases in the order (Bth)_3_ << (Bth)(BimH)_2_ < (BimH)_3_ << (Bth)_2_(BimH) [80,83]. With carboxylic acid or ester groups attached to the benzimidazole rings via alkyl chains (CH_2_)_4_ and (CH_2_)_5_, the CuAAC test reaction was substantially accelerated [(BimC_4_A)_3_, (BimC_3_A)_3_, (BimH)(BimC_5_A)_2_, (BimC_5_A)_3_, (BimC_4_A/Me_2_)_3_, (BimC_4_E)_3_]. On the other hand, (BimC_1_A)_3_ produced one of the worst performing catalysts as the acid group is directly attached to the benzimidazole by a CH_2_-linker. Many of the observed reactions are sensitive towards the choice of pH and type of buffer, which might be due to a change in the rate-limiting step of the catalytic cycle or influences on structure and speciation. The water-soluble ligand (BimC_4_A)_3_ was found to be the most convenient for a variety of substrates in aqueous solutions with only 0.01 to 0.50 mol % copper ions, as these reactions were found to be very fast, high-yielding and insensitive towards a wide range of pH values. On the other hand, tris(2-pyridylmethyl)amine (Py)_3_, the secondary amine H(BimH)_2_ and [Py(BimC_4_A)_2_] were shown to slow down the reaction or have no effect on its rate. The rational explanation for this behaviour together with a mechanistic proposal for CuAAC reactions with this family of ligands is presented in this review’s section on kinetic studies.

**Figure 2:**
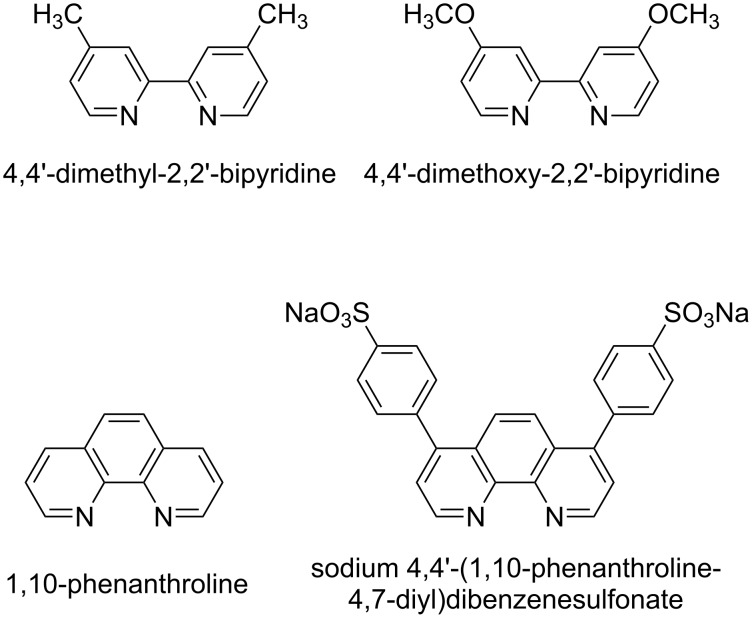
Derivatives of 2,2’-bipyridine and 1,10-phenanthroline, commonly used ligands in CuAAC reactions under Sharpless–Fokin conditions [84].

In one of their first investigations regarding the influence of additives, Fokin and Finn have presented 2,2’-bipyridine and 1,10-phenanthroline derivatives as effective ligands for CuAAC reactions with copper(II) sulfate and sodium ascorbate (Figure 2) [84].

A two- to three-fold increase in the rate of reaction was observed with this class of ligands. Bathophenanthrolinedisulfonate turned out to be an excellent ligand as it is water-soluble and allows for colourimetric detection. A ligand:copper ratio of 2:1 was found to be optimal, and under these conditions the rate order with respect to the complex [CuL_2_] was found to be two. However, if these ligands are added in excess quantities (ligand:metal ratio > 2:1), the reaction is dramatically slowed down or does not work at all. As the rigid chelating ligands of this class bind so strongly to copper(I) ions, they form inhibitory species. Catalysis is usually shut down completely when an excess of 2,2’-bipyridine or 1,10-phenanthroline derivatives is present in the reaction mixture [80]. Although the structural characteristics of the corresponding copper complexes remain elusive, these ligands are frequently applied in CuAAC reactions, especially in macromolecular chemistry [15].

**Scheme 4:**
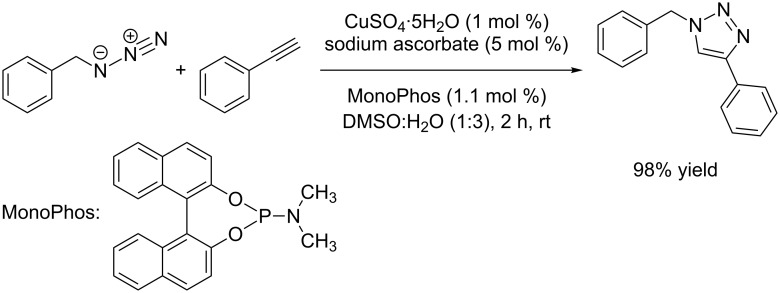
CuAAC reaction with copper(II) precursor salt and rate-accelerating monodentate phosphoramidite ligand MonoPhos [85].

Apart from *N*-donor ligands, the use of phosphorus additives in combination with copper(II) salts has been reported. In 2009, Feringa used phosphoramidite ligands to accelerate CuAAC reactions in aqueous media (Scheme 4) [85]. With the traditional Sharpless–Fokin system copper(II) sulfate pentahydrate (1 mol %) plus ascorbate (5 mol %) as reducing agent, the reaction of phenylacetylene (1.2 equivalents) with benzyl azide gave 98% yield within two hours when the monodentate phosphoramidite ligand MonoPhos (1.1 mol %) was present in the aqueous reaction mixture (DMSO/H_2_O 1:3). In the absence of MonoPhos, the reaction was only completed after 30 hours under otherwise identical conditions (88% yield). The authors suggest a copper acetylide complex to be the active species, but such complexes have neither been isolated nor structurally characterized.

The group of Novák added triphenylphosphine (2 mol %) to the catalyst precursor copper(II) salts with the aim of enabling CuAAC in organic media [86]. In this protocol, the triphenylphosphine additive (2 mol %) does not only act as ligand to increase the solubility of the copper species, but does also fulfil the role of the reducing agent to effect the conversion of Cu(II) to the catalytically active Cu(I) (similar to TCEP as reducing agent in biological applications [45–55]). However, Staudinger side reactions between the azide and the phosphine may take place as well, leading to the formation of the corresponding amine and triphenylphosphine oxide as byproducts [56–58] as well as lack of phosphine ligands for the copper(I) species. With toluene as solvent, only 40% conversion in the reaction of benzyl azide with phenylacetylene was observed after 24 hours at room temperature when copper(II) sulfate pentahydrate (1 mol %) was used as catalyst precursor. However, with copper(II) acetate hydrate (1 mol %), complete conversion was observed within one hour under identical conditions. The authors thus point out the beneficial effect of carboxylate compared to other copper salts. This effect can most probably be explained by the basic character of the carboxylate anion (compared to sulfate, for example), which facilitates the deprotonation of the π-coordinated alkyne substrate in aprotic solvents [87].

#### CuAAC catalysis with mixtures of copper(I) species and ligand precursors

Instead of forming the catalytically active copper(I) species in situ, a copper(I) salt can be added straightaway. Yet, this procedure is much less robust than the use of copper metal or copper(II) salts as precatalysts and usually demands an inert atmosphere and anhydrous solvents. In his seminal publication, Sharpless describes the use of copper(I) iodide, CuOTf·C_6_H_6_ and [Cu(H_3_CCN)_4_][PF_6_]. However, acetonitrile as co-solvent and the presence of a nitrogen base such as triethylamine, *N,N*-diisopropylethylamine (DIPEA) or pyridine are required for these catalyses to work. Sharpless also reports the formation of diacetylenes by alkyne homocoupling as well as bistriazoles and 5-hydroxytriazoles as undesired byproducts. Inert gas conditions and the application of 2,6-lutidine reduce this problem [12].

Amine ligands are still very popular for CuAAC reactions with Cu(I) precursors [15–16]. Triethylamine can be used as ligand for copper(I) ions prepared in situ in aqueous media [88–89], and fulfil the twofold role of base and ligand in organic solvents [90–97]. Although a variety of other tertiary amines such as propylamine [98–99] or tributylamine [100] has been employed, there is no comprehensive study about the influence of the nitrogen substituents. The structure of the catalytically active species is unknown.

**Scheme 5:**
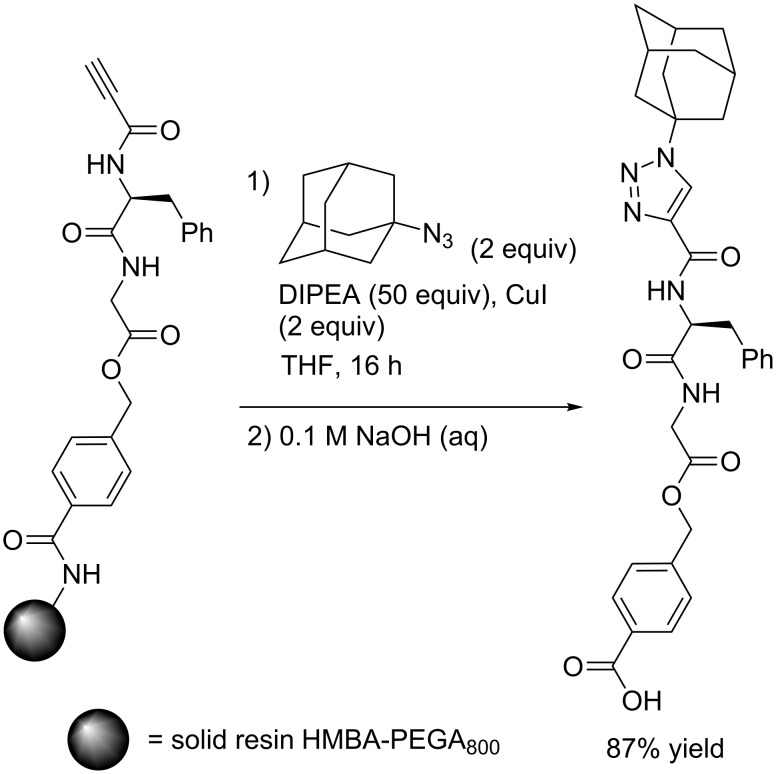
Synthesis of 1-(adamant-1-yl)-1*H*-1,2,3-triazol-4-ylcarbonyl-Phe-Gly-OH by solid-supported Click catalysis and subsequent release of the product from the resin by hydrolysis [8].

For the solid-phase synthesis of peptidotriazoles, the group of Meldal used copper(I) iodide in combination with DIPEA (Scheme 5). The author pointed out that albeit copper(I) iodide was used in stoichiometric amounts (2 equivalents), this was only due to the small scale of the reactions – catalysis was also effected by concentrations as low as 0.01 equivalents of copper(I) iodide [8].

The group of Wong has used copper(I) iodide for the synthesis of triazole-containing saccharides and their attachment to microtiter plates [90]. For the optimization of conditions, the reaction between 2-azidoethyl-β-D-galactopyranoside and *N*-tetradecylpropiolamide was carried out either without addition of a base or in the presence of triethylamine or DIPEA. In the absence of any base, the reaction proceeded very slowly. This is most probably due to the use of an aprotic solvent, which does not facilitate the deprotonation of the alkyne substrate. Usually when copper(II) precursors are used, protic solvents and basic reducing agents such as sodium ascorbate are present in the reaction mixture. Heaney has pointed out that ascorbate is needed in excess as it acts as a base in the deprotonation of the π-complexed alkyne in organic media [87]. Moreover, it is speculated that even in the absence of a basic reducing agent, e.g. by use of TCEP, the copper(I) ions formed in situ from copper(II) are more reactive towards the alkyne than copper(I) bromide or iodide, as the latter contain very stable cluster structures so that a minimum concentration of acetylide is needed for the reactive copper acetylide complex to form [19].

However, when Wong used copper(I) iodide with triethylamine and acetonitrile as solvent, the reaction of 2-azidoethyl-β-D-galactopyranoside with *N*-tetradecylpropiolamide afforded only traces of product. On the other hand, the addition of DIPEA to the reaction mixture in acetonitrile gave the desired product in 38% yield after 18 hours. In toluene, the reaction with triethylamine afforded 65% conversion and 85% with DIPEA. These findings illustrate the high sensitivity of the catalysis towards the choice of additives and reaction conditions.

Generally, DIPEA [8,90,101–108] and 2,6-lutidine [12,64,109–116] seem to be best suited for CuAAC and an excess of base is favourable to the reaction as well. Although these conditions are termed “ligand-free” it is obvious that solvent molecules such as acetonitrile as well as the nitrogen base will coordinate to the copper(I) ions. These coordinating additives ameliorate the catalytic performance by preventing oxidation and disproportionation processes [18] as well as the formation of unreactive polynuclear copper(I) aggregates (Scheme 19) [42,63].

Only in (aprotic) organic media are amine additives also essential in their role as base to deprotonate the alkyne substrate, whereas the formation of the corresponding copper(I) acetylides in aqueous media is so facile that it even occurs in strongly acidic solutions [117].

Sulfur-based ligands are rarely used for CuAAC catalysis. However, the copper(I) bromide dimethyl sulfide complex [CuBr·SMe_2_]_2_ is well soluble in organic media, commercially available and shows good catalytic performance [118–119]. For example, the group of Nakamura used [CuBr·SMe_2_]_2_ as catalyst in THF to synthesize triazole-linked DNA analogues either in solution or on solid support [120]. Of the variety of sulfur-containing compounds tested by the group of Fu, thioanisole turned out to be a good ligand for CuAAC catalysis. With this ligand (30 mol %) and copper(I) bromide (5 mol %) the reaction of benzyl azide with phenylacetylene is completed within 10 minutes in aqueous solution at room temperature under aerobic conditions [118].

Phosphorus ligands in combination with copper(I) salts have been reported by the group of Novák [86]. In the absence of any additives, copper(I) iodide, bromide, chloride or cyanide salts did not effect any conversion in the reaction mixture of benzyl azide and phenylacetylene in toluene at room temperature. However, by addition of triphenylphosphine 41% conversion was observed within three hours in the case of copper(I) iodide. By addition of potassium acetate as base, this reaction was substantially accelerated to give 75% conversion after three hours. Employing other carboxylate bases such as sodium propionate, butyrate and caprylate gave even better results. By using copper(I) carboxylate salts, the addition of an external base can be circumvented. Excellent results were obtained when the reaction between benzyl azide and phenylacetylene in toluene was carried out in the presence of a reagent mixture of triphenylphosphine and copper(I) acetate (full conversion within one hour at room temperature). The effect of the phosphine ligand is generally attributed to an increase in the copper(I) salt’s solubility in organic media.

Feringa et al. used copper(I) salts in combination with phosphoramidite ligands such as MonoPhos. With copper(I) chloride (1 mol %) and MonoPhos ligand (1.1 mol %) the test reaction of benzyl azide with phenylacetylene gave 99% yield after one hour in an aqueous medium (DMSO:H_2_O 1:3) [85].

#### CuAAC catalysis with molecularly defined copper(I) complexes as (pre-)catalysts

Only recently, the potential of copper(I) acetate as heterogeneous catalyst for CuAAC reactions has been investigated by the group of Wang [121]. The authors of this study had noticed that the Cu–Cu distance found in polymeric copper(I) acetate [(CuH_3_CCO_2_)_2_]*_n_* (2.556 Å) [122–124] was in the same range as the distance for effective CuAAC catalysis with dinuclear copper(I) complexes as calculated in a DFT study by Ahlquist and Fokin [125] (transition states with Cu–Cu distances of 2.54 Å for L = X = chloride and 2.64 Å for L = acetylide). They tested copper(I) acetate in the CuAAC model reaction of benzyl azide with phenylacetylene and reported an excellent performance of this heterogeneous catalyst in a variety of solvents at room temperature under aerobic conditions. At the beginning of each reaction, the authors observed a bright yellow colour, which might be due to the formation of transient copper(I) acetylide species [126–127]. And indeed did the isolation of this yellow compound in the absence of benzyl azide show that its elemental analysis and IR spectra are the same as for commercially available phenylethynyl copper(I) PhC≡CCu.

**Scheme 6:**
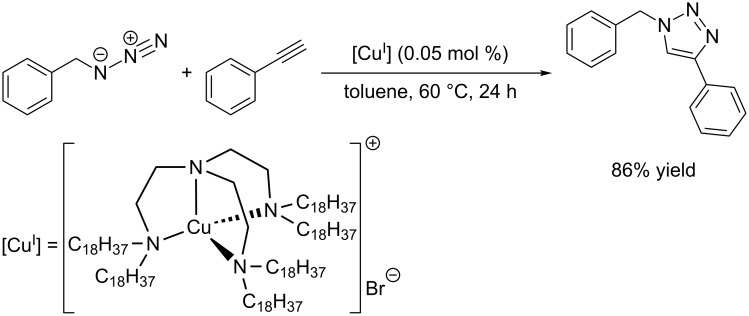
CuAAC reaction with re-usable copper(I)-tren catalyst [129].

In 2004, the group of Vincent first presented tris(2-dioctadecylaminoethyl)amine (C18_6_tren), a sterically crowded tripodal ligand [128]. The corresponding copper(I) complex [Cu(C18_6_tren)]Br does not have to be prepared in situ, but can be isolated and handled in air. This is one of the few examples, where the copper(I) (pre-)catalyst complex is molecularly defined and characterized. As triazoles are more polar than the catalyst complex, CuAAC reactions can be conveniently carried out in toluene or *n*-octane, where the triazole product precipitates and can be easily isolated by filtration under aerobic conditions. On the other hand, the filtrate containing the catalyst can be re-used. For example, the model reaction of benzyl azide with phenylacetylene carried out in toluene at 60 °C with 0.05 mol % [Cu(C18_6_tren)]Br affords 86% yield after 24 hours (Scheme 6) [129].

**Scheme 7:**
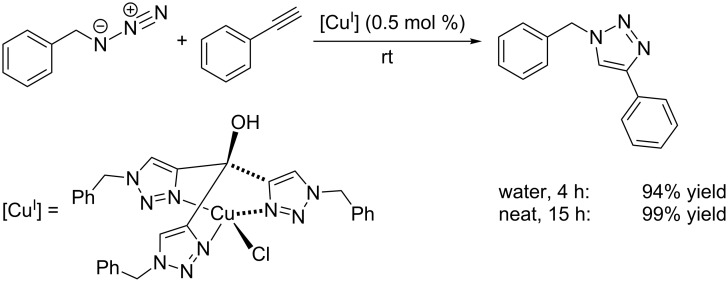
CuAAC test reaction with chlorido[tris(1-benzyl-1*H*-1,2,3-triazol-4-yl)methanol-κ^3^*N^3^*]copper(I) and assumed structure of the (pre-)catalyst complex [130].

**Scheme 8:**
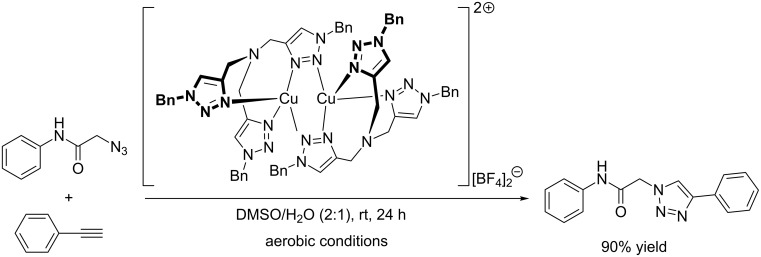
CuAAC model reaction with [Cu_2_(*μ*-TBTA-κ^4^*N^2^*,*N^3^*,*N^3^’*,*N^3^’’*)_2_][BF_4_]_2_ [131].

Another water-soluble derivative, tris(1-benzyl-1*H*-1,2,3-triazol-4-yl)methanol, was found to be an excellent ligand for the preparation of air-stable copper(I) complexes, which are highly active in CuAAC reactions on water or under neat conditions (Scheme 7) [130]. Albeit no single crystals of the copper(I) complex shown in Scheme 7 could be grown, NMR measurements as well as a single crystal X-ray structure of an analogous C18_6_tren-complex with CuCl_2_ hint at the formation of a 1:1 complex between this ligand and copper(I) chloride. However, it is questionable whether this mononuclear species or higher aggregates of the latter are the catalytically active species in the CuAAC reaction, especially in the light of Donnelly’s report on a dinuclear TBTA complex (Scheme 8) [131].

In 2008, the group of Donnelly was able to crystallize a dinuclear copper(I)-TBTA complex from TBTA and tetrakis(acetonitrile)copper(I) tetrafluoroborate under anaerobic conditions in acetonitrile [131]. The single crystal X-ray structure shows a dinuclear dication [Cu_2_(μ-TBTA-κ^4^*N^2^*,*N^3^*,*N^3^’*,*N^3^’’*)_2_]^2+^, in which the coordination geometry at each copper(I) centre is a distorted tetrahedron. In contrast to the structural hypothesis by Sharpless and Fokin (vide supra) [64], the central nitrogen of the tertiary amine does not coordinate to either copper(I) ion in the solid state. Instead the copper ions are bound to three proximal and one medial nitrogen atom each, i.e. one triazole ring in each TBTA molecule bridges the two copper ions as shown in Scheme 8. The air-sensitive salt [Cu_2_(μ-TBTA-κ^4^*N^2^*,*N^3^*,*N^3^’*,*N^3^’’*)_2_][BF_4_]_2_ was shown to be an effective catalyst in the CuAAC test reaction of phenylacetylene with 2-azido-*N*-phenylacetamide even under aerobic conditions. With tetrakis(acetonitrile)copper(I) hexafluorophosphate in the absence of TBTA, no reaction was observed under identical conditions; only the addition of TBTA triggered CuAAC catalysis. Albeit the crystal structure of the [Cu_2_(μ-TBTA-κ^4^*N^2^*,*N^3^*,*N^3^’*,*N^3^’’*)_2_][BF_4_]_2_ precatalyst has been solved, the structural characteristics of the active species in solution remain unknown.

**Scheme 9:**
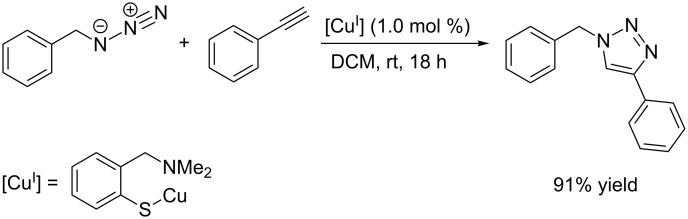
Application of a (2-aminoarenethiolato)copper(I) complex as homogeneous catalyst for the CuAAC test reaction [132].

A homogeneous catalyst for CuAAC reactions in organic solvents was presented by van Koten et al. in 2009 [132]. The (2-aminoarenethiolato)copper(I) complex reported in their work is an efficient catalyst for CuAAC reactions with a variety of substrates in dichloromethane or acetonitrile at room temperature. For example, the standard test reaction between benzyl azide and phenylacetylene in dichloromethane gives 91% yield within 18 hours (Scheme 9). As copper(I) thiolate complexes show a high tendency to form aggregates [133–136], speciation and nuclearity of the catalytically active species remain unknown.

**Scheme 10:**
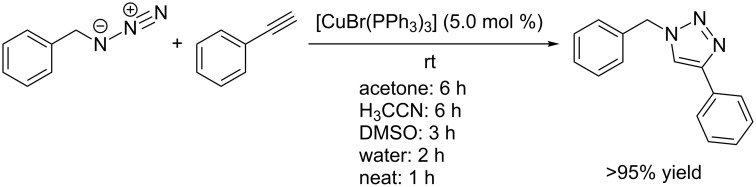
Application of [CuBr(PPh_3_)_3_] as homogeneous catalyst for the CuAAC test reaction of benzyl azide with phenylacetylene under homogeneous conditions in acetone, acetonitrile and DMSO, and as heterogeneous catalyst in water or under neat conditions [138].

Copper(I) complexes with phosphorus ligands, for example the commercially available air-stable salts [CuBr(PPh_3_)_3_] and {CuI[P(OEt)_3_]}, are also well soluble in organic solvents and do thus allow for homogeneous CuAAC reactions. They were first applied in the synthesis of neoglycoconjugates in the presence of DIPEA or DBU under microwave irradiation in toluene solution [137]. However, a recent study by Díez-González has shown, that neither irradiation nor any additive is necessary for [CuBr(PPh_3_)_3_] to act as an effective CuAAC precatalyst [138]. In a solvent mixture of water and *tert*-butanol, the test reaction of benzyl azide with phenylacetylene proceeded within two hours to give 95% yield when 5 mol % of [CuBr(PPh_3_)_3_] were present. Acetone, DMSO and acetonitrile also turned out to be suitable solvents for this catalyst system (Scheme 10). The catalyst loading for this reaction can be decreased to 500 ppm and full conversion is still reached within 24 hours under neat conditions.

In this study, Díez-González also attempted the in situ preparation of the organoazide from the corresponding organic bromide and sodium azide [138]. Albeit the organoazide was formed smoothly in DMSO or acetone, the cycloaddition reaction with phenylacetylene did not take place. A control experiment with benzyl azide, phenylacetylene and addition of sodium bromide showed that this salt exerts an inhibitory effect on CuAAC catalysis with [CuBr(PPh_3_)_3_]. Only by applying high catalyst loadings and long reaction times could conversion to the triazole product be observed. However, this effect was not found when water was used as reaction medium. It is supposed that bromide ions can bind strongly to copper(I) centres in organic media, but due to a tight layer of solvent molecules they cannot do so in aqueous solution. Thus, the CuAAC reaction with in situ generated azides could only be carried out in water as reaction medium. {The detrimental influence of halide ions on CuAAC reactions has also been reported by Finn et al., who observed an inhibitory effect of chloride ions in aqueous CuAAC reaction mixtures with more than 0.5 M of sodium chloride when using phosphate-buffered saline [42,71]. With copper(I) iodide as catalyst, the formation of 5-iodotriazoles as byproducts has been observed under various conditions [139–141].}

Chen et al. presented [Cu(PPh_3_)_2_]NO_3_ as an effective CuAAC catalyst in toluene, water or under neat conditions. With 0.5 mol % of this catalyst, the test reaction of phenylacetylene with benzyl azide in toluene proceeds within 40 minutes at room temperature to give 96% yield. Under neat conditions, the amount of catalyst can be lowered to 50 ppm and the reaction still gives 81% yield after 24 hours [142]. Recently, it has been shown that the phenanthroline complex [Cu(phen)(PPh_3_)_2_]NO_3_ outperforms their original catalyst [Cu(PPh_3_)_2_]NO_3_. With 2 mol % [Cu(phen)(PPh_3_)_2_]NO_3_ the model reaction of phenylacetylene and benzyl azide under neat conditions is finished after three minutes and gives 97% yield [143].

The group of Novák did not only use various copper(I/II) salts in combination with triphenylphosphine in order to carry out CuAAC reactions in organic solvents, but also assessed the catalytic performance of pre-formed copper(I) butyrate complex [Cu(C_3_H_7_COO)(PPh_3_)_2_], which showed the best activity among the copper(I) phosphine complexes tested [86]. For example, with 500 ppm of this catalyst, complete conversion in the standard test reaction between benzyl azide and phenylacetylene at room temperature in dichloromethane was observed after two hours.

**Figure 3:**
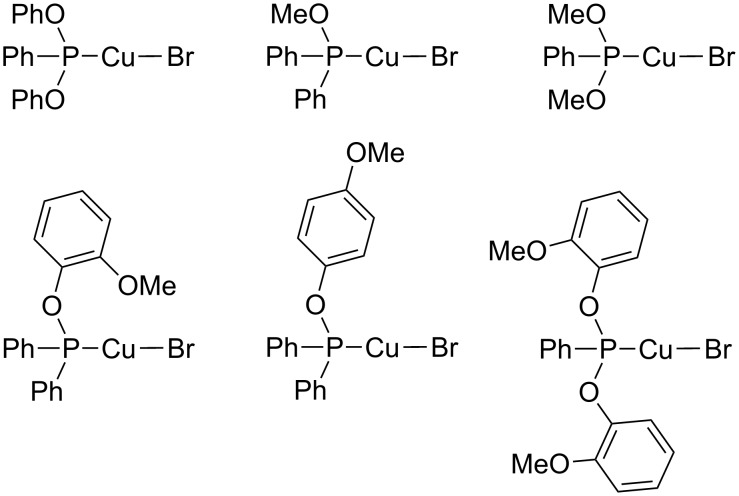
Phosphinite and phosphonite copper(I) complexes presented by Díez-González [144].

In 2011, the group of Díez-González introduced phosphinite and phosphonite copper(I) complexes (Figure 3) as novel molecularly defined precatalysts for CuAAC reactions under Click conditions [144].

These copper(I) complexes can be handled under aerobic conditions and have been fully characterized including single crystal X-ray structures, which show a cubane-like [Cu_4_Br_4_] scaffold. Their main advantages are the ease of preparation, tolerance towards water and air, and that they are molecularly defined so that their characteristics can be tuned according to specific demands. In CuAAC reactions with these complexes, no additives are needed. For example, the model reaction of phenylacetylene with benzyl azide in the presence of 0.5 mol % {CuBr[PPh_2_(OPh-2-OMe)]} (Figure 3, second row, left) in water proceeds at room temperature to give >95% conversion after three hours.

In organometallic catalysis, *N*-heterocyclic carbene ligands (NHCs) have superseded phosphine ligands in many fields of application [145]. It was thus only a matter of time until NHC-copper complexes were first employed in CuAAC reactions. In 2006, the group of Nolan reported the application of air-stable complexes [(NHC)CuX] as efficient catalysts for CuAAC reactions with terminal and internal alkynes in aqueous solution or under neat conditions (Table 2) [146–147]. Also, the azide substrates could be prepared in situ by reaction of the corresponding bromide with sodium azide. For example, the reaction of benzyl bromide with sodium azide and phenylacetylene in the presence of 2 mol % [(SIMes)CuBr] in water gave 86% 1-benzyl-4-phenyl-1*H*-1,2,3-triazole within 18 hours at room temperature. However, in organic solvents such as THF, dichloromethane or *tert*-butanol, only poor conversions were observed with these [(NHC)CuX] catalysts.

**Table 2:** CuAAC catalysts of type [(NHC)CuX] and their performance in the CuAAC test reaction [147].

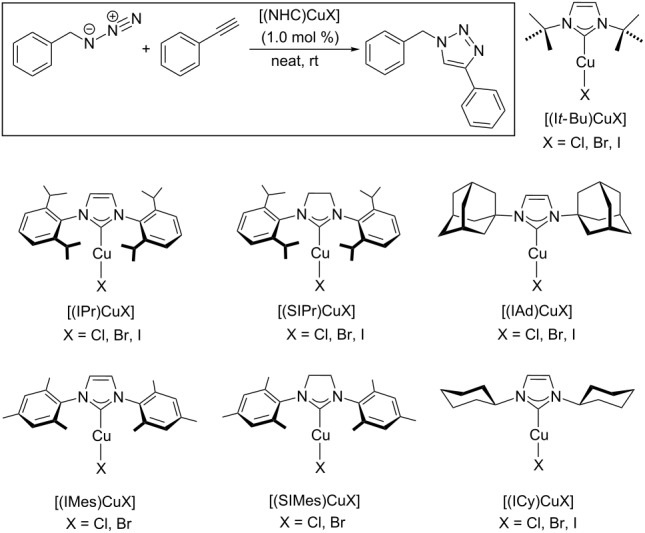					

[(NHC)CuX]	reaction time	conv. [%]	[(NHC)CuX]	reaction time	conv. [%]

[(IPr)CuCl]	24 h	88	[(IMes)CuCl]	1 h	>99
[(IPr)CuBr]	24 h	63	[(IMes)CuBr]	30 min	>99
[(IPr)CuI]	4 h	95	[(SIMes)CuCl]	20 min	>99
[(SIPr)CuCl]	15 h	99	[(SIMes)CuBr]	20 min	>99
[(SIPr)CuBr]	10 h	96	[(ICy)CuCl]	10 min	>99
[(SIPr)CuI]	6 h	>99	[(ICy)CuBr]	1 h	>99
[(IAd)CuCl]	20 min	>99	[(ICy)CuI]	2 h	87
[(IAd)CuBr]	10 min	>99	[(I*t*-Bu)CuCl]	1 h	>99
[(IAd)CuI]	10 min	>99	[(I*t*-Bu)CuBr]	30 min	>99
			[(I*t*-Bu)CuI]	2 h	>99

Nolan et al. also report on the possibility of using [(SIPr)CuCl] as a latent catalyst [148]. With DMSO as solvent, complex [(SIPr)CuCl] (2 mol %) does not facilitate the cycloaddition reaction of benzyl azide and phenylacetylene within one week. However, the latent catalyst could be activated by adding water and heating the reaction mixture to 60 °C for one hour, whereupon 99% conversion was observed.

**Scheme 11:**
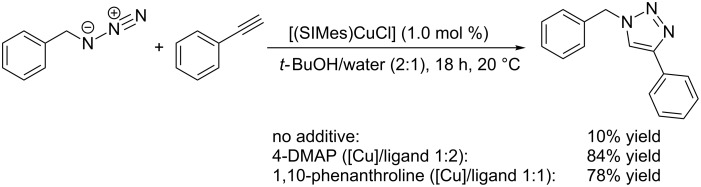
Effect of additives on the CuAAC test reaction with [(SIMes)CuCl] [149].

In 2009, Gautier et al. reported that CuAAC catalysis with [(SIMes)CuCl] can be notably improved by addition of aromatic nitrogen donor ligands [149]. For example, fast homogeneous catalysis in water/alcohol solvent mixtures is possible with [(SIMes)CuCl] in the presence of 4-DMAP or 1,10-phenanthroline (Scheme 11).

The catalyst complex [(SIMes)CuCl(phen)] could be isolated as red crystals and a single crystal X-ray structure shows a distorted tetrahedral coordination geometry at the metal centre. Peak broadening indicative of ligand exchange was observed on the NMR time scale and the association constant of phenanthroline was determined to be approximately *K* = 250 M^−1^. This relatively low value suggests that the phenanthroline ligand only binds weakly to the (SIMes)Cu(I)-fragment and can be easily replaced by the CuAAC’s substrates. In an extended screening of *N*-donor additives, the efficiency in the CuAAC test reaction improved in the following order of ligands: neocuproine < 4,7-dimethoxy-1,10-phenanthroline < bathophenanthroline < 1,10-phenanthroline < 4,7-dichloro-1,10-phenanthroline [150].

In 2006, the group of Nolan introduced a new family of monocationic copper(I) NHC-complexes of general formula [(NHC)_2_Cu]X (X = BF_4_ or PF_6_) as catalysts for hydrosilylation reactions of aldehydes, ketones and esters [151–152]. These complexes also display very high catalytic activity in CuAAC reactions in water (Table 3), under neat conditions or as homogeneous catalysts in acetonitrile solution [153].

**Table 3:** Performance of different [(NHC)_2_Cu]X (X = BF_4_ or PF_6_) precatalysts in the CuAAC test reaction of benzyl azide with phenylacetylene in water at room temperature [153].

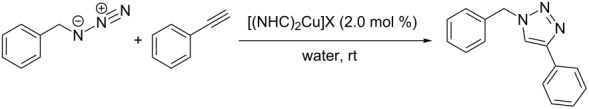					

[(NHC)_2_Cu]X	reaction time	conv. [%]	[(NHC)CuX]	reaction time	conv. [%]

[(IPr)_2_Cu]PF_6_	18 h	71	[(IPr)_2_Cu]BF_4_	8 h	100
[(SIPr)_2_Cu]PF_6_	5 h	100	[(SIPr)_2_Cu]BF_4_	5 h	100
[(IMes)_2_Cu]PF_6_	6 h	100	[(IMes)_2_Cu]BF_4_	6 h	100
[(SIMes)_2_Cu]PF_6_	18 h	5	[(SIMes)_2_Cu]BF_4_	18 h	13
[(ICy)_2_Cu]PF_6_	1.5 h	99	[(ICy)_2_Cu]BF_4_	5 h	95
[(IAd)_2_Cu]PF_6_	5 h	100	[(IAd)_2_Cu]BF_4_	3 h	100
[(I*t*-Bu)_2_Cu]PF_6_	18 h	76	[(I*t*-Bu)_2_Cu]BF_4_	18 h	35

[(ICy)_2_Cu]PF_6_ turned out to be the most efficient precatalyst (Table 3). It was thus tested in a variety of solvents and under neat conditions (Table 4). In alcoholic solvents, the reaction with [(ICy)_2_Cu]PF_6_ is sluggish, but in organic solvents such as acetone or acetonitrile, the CuAAC reaction proceeds even faster than in aqueous solution (Table 4). Neat conditions were found to be optimal as conversion was completed within five minutes with only 0.5 mol % [(ICy)_2_Cu]PF_6_. As the amount of catalyst was lowered to 50 ppm, still 80% conversion was observed within 48 hours at room temperature.

**Table 4:** Application of [(ICy)_2_Cu]PF_6_ in different organic solvents, water and under neat conditions [153].

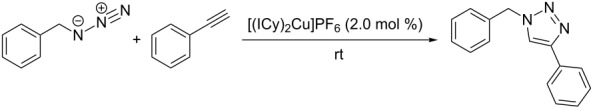							

solvent	neat^a^	water	DMSO	DMF	THF	acetone	acetonitrile

time [min]^b^	5	90	120	90	60	30	12

^a^The reaction under neat conditions was carried out with only 0.5 mol % [(ICy)_2_Cu]PF_6_. ^b^Reaction time until full conversion was observed by GC or NMR measurements.

**Scheme 12:**
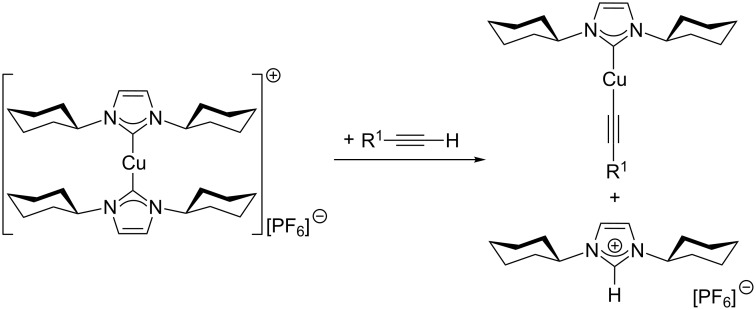
Initiation of the catalytic cycle by formation of the copper acetylide intermediate from [(ICy)_2_Cu]PF_6_ and the alkyne substrate [153].

Mechanistically, one of the two NHC ligands is supposed to play an active role in the catalytic cycle. After its dissociation from the precatalyst [(NHC)_2_Cu]X, the free *N*-heterocyclic carbene can act as a base in the deprotonation of the alkyne substrate (Scheme 12). This protonation of the free *N*-heterocyclic carbene by the alkyne substrate with formation of the corresponding azolium salt is highly favoured, as copper-coordinated alkynes are much more acidic than imidazolium or imidazolinium cations. For example, the p*K*_a_ value of copper-coordinated propyne was calculated to be around 15 [13], whereas *N*,*N*-diarylimidazolium and *N*,*N*-diarylimidazolinium chloride salts have p*K*_a_ values of about 19.8 to 21.1 and 20.7 to 21.5, respectively, for example p*K*_a_ [1,3-bis(2,6-diisopropylphenyl)imidazolium chloride] = 21.1 ± 0.5 and p*K*_a_ [1,3-bis(2,6-diisopropylphenyl)imidazolinium chloride] = 21.5 ± 0.5 in aqueous solution at 25 °C [154]. In contrast to free *N*-heterocyclic carbenes, azolium cations cannot compete for free coordination sites at the copper(I) centres so that the formation of copper acetylide complexes is greatly facilitated. This “built-in” base, the irreversible deprotonation of the alkyne under the reaction conditions of this protocol and the lack of species that can compete with the substrates for free coordination sites at the copper(I) centre are probably the main factors why some complexes of type [(NHC)_2_Cu]X are catalytically more active than the [(NHC)CuX] family.

### Mechanistic studies

#### Mononuclear mechanistic proposal

**Scheme 13:**
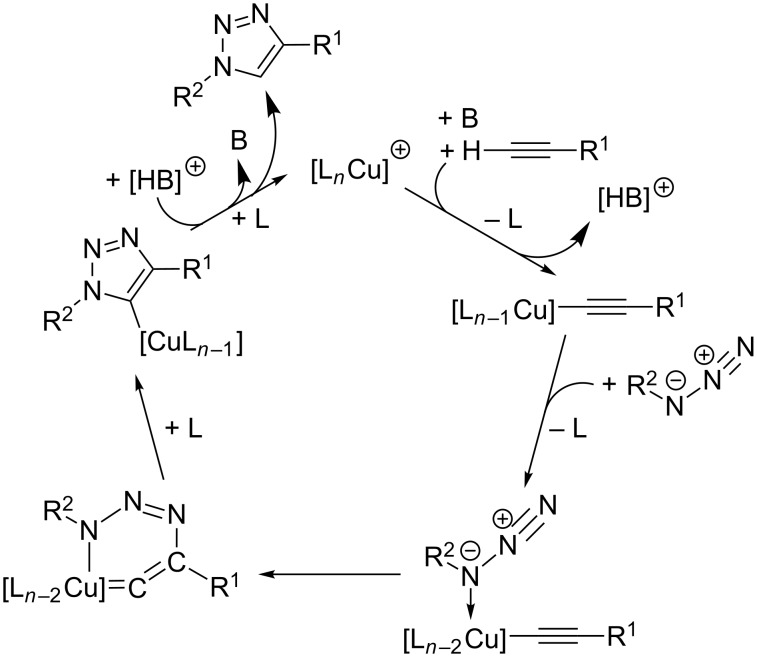
Early mechanistic proposal by Sharpless [12,42].

In his seminal publication, Sharpless disclosed a first proposal for the CuAAC’s mechanism [12]. As shown in Scheme 13, the alkyne substrate is deprotonated and σ-coordinates to the copper(I) centre of the active catalyst. If there is another free coordination site, the azide can coordinate to this copper(I) ion as well. The C–N bond is formed concomitantly to the formation of a double bond between the copper ion and the C1 atom of the acetylide. This unusual six-membered copper(III) metallacycle then undergoes a transannular ring contraction to give the copper triazolide. The latter can be protonated to release the triazole product.

#### Excursus: copper-catalyzed cycloaddition reactions of 1-haloalkynes and internal alkynes

Mononuclear mechanistic proposals are also worth mentioning in the context of cycloaddition reactions of haloalkynes and internal alkynes with organoazides to give 1,4,5-trisubstituted 1,2,3-triazoles.

In 2005, the group of Rutjes reported the synthesis of 5-bromo-1,4-disubstituted-1,2,3-triazoles starting from 1-bromoalkynes [155]. In their optimized procedure, the bromoalkyne reacts with an azide in the presence of 5 mol % copper(I) iodide and 5 mol % copper(II) acetate in THF at 50 °C to give the 5-bromo-1,4-disubstituted-1,2,3-triazoles with small quantities of the 5-iodo-1,4-disubstituted-1,2,3-triazole as byproduct. Obviously, the alkynyl halide substrate can exchange its halide group for other halide ions present in the reaction mixture, which means that the formation of this byproduct can be prevented by using copper(I) bromide instead of the iodide salt.

This reactivity of 1-haloalkynes is in accordance with the observation that 5-iodo-1,2,3-triazoles are formed as byproducts, when CuI is used as catalyst in CuAAC reactions of azides and terminal alkynes as has been reported by Pérez-Castro [139] and Tanaka [140]. It has been proposed that the iodide ion can act as μ_2_-bridging ligand so that polynuclear copper(I) acetylide complexes are formed, which are less active in the catalytic cycle for the formation of 5*H*-1,2,3-triazoles. Fokin suggests that under certain conditions, the formation of 1-iodoalkynes is facilitated in these aggregates [42]. In accordance with Rutjes’ report on the reactivity of pre-formed 1-haloalkynes [155], these 1-iodoalkynes formed in situ can analogously react to form the corresponding 5-iodotriazoles. Dzyuba et al. managed to influence the incorporation of iodide in CuAAC reactions of terminal alkynes mediated by CuI (1.0 equivalent) by adequate choice of the base present in the reaction mixture [141]. With a ratio of alkyne/azide/DMAP/CuI = 1.0/3.0/0.3/1.0, they obtained 96% of the 5-iodotriazole and 4% of the Glaser coupling product. Dzyuba has also challenged the idea that 1-haloalkynes need to be formed as intermediates. Instead, he proposes a dinuclear mechanism very similar to the mechanistic pathway shown in Scheme 22 leading to the same copper triazolide complex, which does only in the presence of DMAP take up a formal “I^+^” ion to give the 5-iodotriazole product. Although DMAP must play an eminent role in this context, the redox chemistry leading to the formation of an “I^+^” equivalent was not discussed by these authors [141].

**Scheme 14:**
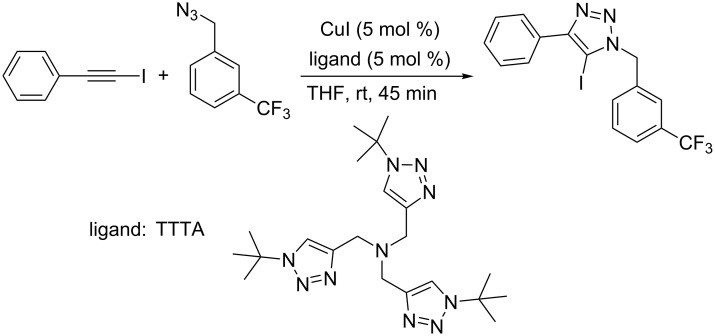
Chemoselective synthesis of a 5-iodo-1,4-disubstituted 1,2,3-triazole [156].

In order to further ameliorate the preparation of 5-iodo-1,4-disubstituted-1,2,3-triazoles, the groups of Sharpless and Fokin devised a new protocol starting from 1-iodoalkynes [156]. The great advantage compared to the procedures of Wu [157], Hsung [158] and Li [159] is that neither reactive electrophilic halogenating agents (such as NBS or iodine monochloride) nor stoichiometric amounts of copper salts need to be employed. Instead the presence of amine bases such as triethylamine or tris[(*tert*-butyl)triazolylmethyl]amine (TTTA) greatly accelerates the highly selective formation of the 5-iodo-1,4-disubstituted-1,2,3-triazoles (Scheme 14).

**Scheme 15:**
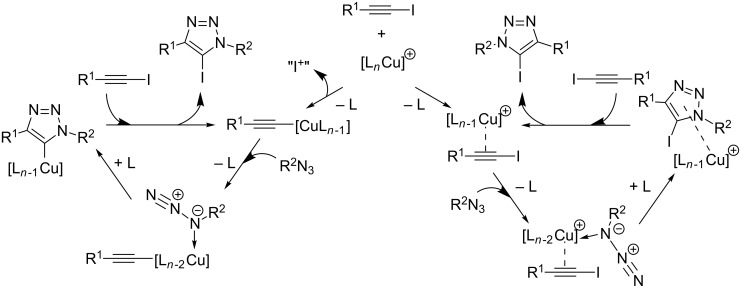
Mechanistic proposals for the copper-catalyzed azide–iodoalkyne cycloaddition [156].

Despite the many similarities to the CuAAC reaction of terminal alkynes, the mechanism of the copper(I)-catalyzed azide–iodoalkyne cycloaddition needs to be significantly different from the CuAAC’s mechanistic pathway shown in Scheme 22. Two catalytic cycles are currently discussed [155–156]: on the one hand, the formation of a copper(I) acetylide intermediate by copper–halogen exchange and trapping of the triazolide with “I^+^” provided by ligand exchange with the 1-iodoalkyne (left side in Scheme 15); on the other hand, the copper(I) centre might only serve to activate the iodoalkyne by π-coordination, so that the cycloaddition process can then take place in the catalyst’s coordination sphere (right side in Scheme 15). The latter pathway seems more probable, as even in protic media such as ethanol and water, only 5-iodo-1,4-disubstituted-1,2,3-triazoles are formed, which means that protonation of the copper(I) triazolide intermediate does not take place. However, exchange of the halide group when 1-bromoalkynes are reacted with azides in the presence of copper(I) iodide [155] as observed by Rutjes et al. cannot be explained by this pathway.

**Scheme 16:**
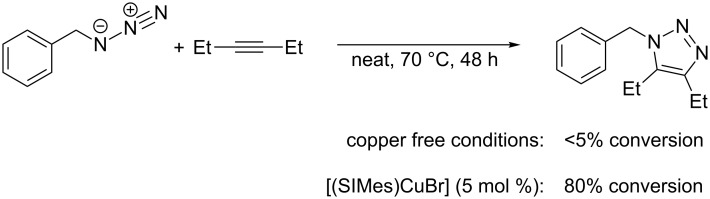
1,3-Dipolar cycloaddition of 3-hexyne catalyzed by [(SIMes)CuBr] [146].

In 2006, the group of Nolan presented the synthesis of 1,4,5-trisubstituted-1,2,3-triazoles by cycloaddition reactions between internal alkynes and azides using catalysts of general formula [(NHC)CuX] [146]. This unprecedented reactivity was tested with 3-hexyne and benzyl azide under neat conditions at 70 °C with 5 mol % [(SIMes)CuBr] as catalyst (Scheme 16).

**Scheme 17:**
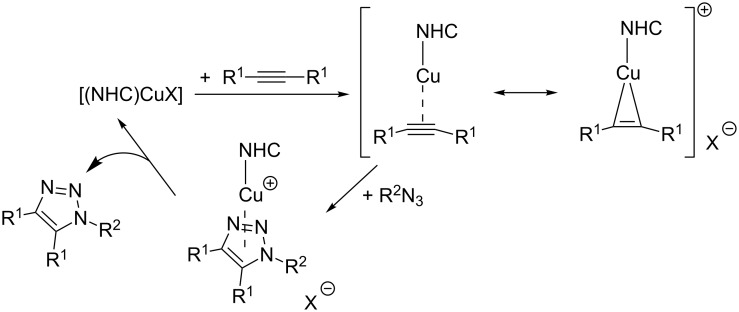
Mechanistic picture for the cycloaddition of internal alkynes catalyzed by NHC-copper(I) complexes as proposed by Nolan [146].

As for the mechanism of this transformation, Nolan proposes that the strong σ-donor NHC ligand facilitates the formation of a copper(I) π-complex with significant π-backbonding, and thus activates the alkyne for the cycloaddition reaction. Based on DFT calculations, the mechanism displayed in Scheme 17 was proposed, which is in analogy to the catalytic cycle presented on the right side of Scheme 15.

To our knowledge, no detailed mechanistic investigations on the subject of CuAAC reactions with internal alkynes have been reported in literature. As a consequence, this specific reactivity will not be further discussed in this review article.

#### From mononuclear to dinuclear mechanistic proposals: computational and experimental evidence

The groups of Fokin and Finn have tested different heterocyclic chelating ligands for CuAAC by fluorescence quenching in the reaction of dansyl azide fluorophore and dabsyl alkyne, and carried out kinetic measurements with a bis(bathophenanthrolinedisulfonate)copper(I) complex formed in situ from copper(II) sulfate and disodium bathophenanthrolinedisulfonate in the presence of sodium ascorbate as reducing agent [84]. The authors found that the rate law of the reaction was second order with respect to the concentration of the copper(I) complex. As a consequence, they suggested two copper centres to be required for catalytic turnover.

Mechanistic studies for the “ligand-free” CuAAC followed [160]. The reaction of benzyl azide with phenylacetylene in dimethyl sulfoxide/water in the presence of copper(II) sulfate pentahydrate and an excess of sodium ascorbate was monitored by taking aliquots at intervals from the reaction mixture by an automated liquid handler under inert gas atmosphere and subsequent LC–MS analysis. The authors found the reaction to be second order in the concentration of copper(I) ions under catalytic conditions. They also reported that the rate of reaction increases more slowly than suggested for rising copper concentrations, which hints at the formation of aggregates at high metal concentrations. This aggregation is prevented by addition of the fully deuterated product 1-benzyl-4-phenyl-1*H*-1,2,3-triazole so that a clean second order dependence on the copper concentration was observed. With excess copper concentrations, the rate law was found to be between first and second order in the concentration of alkyne. The rate order of 1.3 ± 0.2 may either suggest that two pathways are involved with participation of one or two alkyne molecules in the rate-determining step, respectively. Alternatively, there could be only one pathway including two acetylenes in the rate-determining step, which is inhibited by higher concentrations of the alkyne. The latter proposition is supported by the fact that commercially available copper acetylides are catalytically inactive, probably because they are coordinatively saturated by the preferentially bound acetylide, and binding of the azide substrate is inhibited [18]. The assumption that excess quantities of azide inhibit CuAAC catalysis was later corrected [81], since the inhibitory effect was caused by trace impurities in commercially available benzyl azide and not observed when the azide was freshly distilled prior to use. All in all, it is an essential prerequisite for CuAAC catalysis to have labile ligands on the copper(I) centre that can easily dissociate to open free coordination sites for the substrates.

**Scheme 18:**
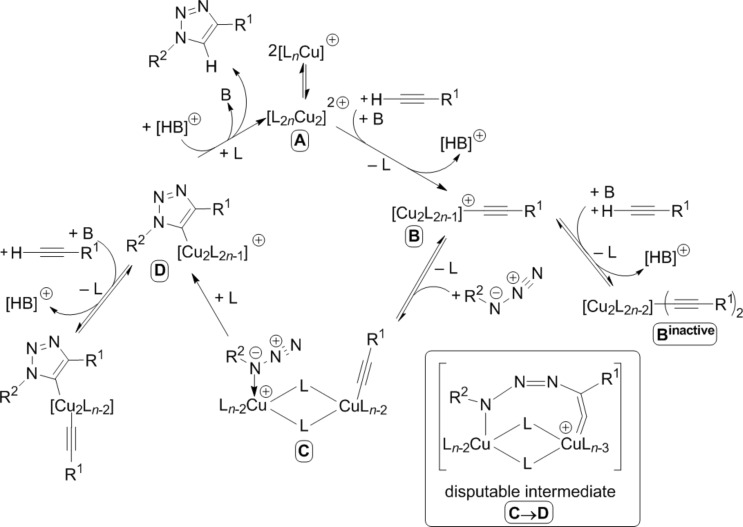
Catalytic cycle of the CuAAC reaction on the basis of the proposed mechanistic scheme by Fokin and Finn in 2005 [160] and a disputable intermediate for the first C–N bond-forming step as formulated in a review article by van Maarseeven [18].

Based on these findings and the observation of polynuclear copper(I) alkyne complexes by the groups of Mykhalichko and García-Granda [117,161], Fokin and Finn presented their mechanistic understanding of the CuAAC reaction as summarized in Scheme 18 [160].

However, the precise structure of the putative dinuclear copper catalyst remains unknown [18] and is thus abbreviated by [L_2_*_n_*Cu_2_]^2+^ (**A**; all charges and numbers of ligands have been consistently accounted for in a stoichiometric “book-keeping” fashion, but are not meaningful, as anionic ligands might also be present in the reaction mixture). Under various reaction conditions, it has been observed that the CuAAC reaction mixtures show a transient yellow colour upon addition of the copper catalyst (precursor) to the substrates’ solution. This colour has been attributed to the formation of polynuclear organocopper species [42,87,121,162–163]. As the yellow colour fades when the reaction progresses, Fokin suggests that the “catalyst is undergoing reorganization while the catalytic cycle is turning over during the initial” period “after the start of the reaction” [42]. This means that speciation and nuclearity of the copper(I) complexes present in the reaction mixture as well as the concentration of the active catalyst might change at the beginning of the experiment until steady state is reached. Such processes hamper the interpretability of kinetic measurements.

**Figure 4:**
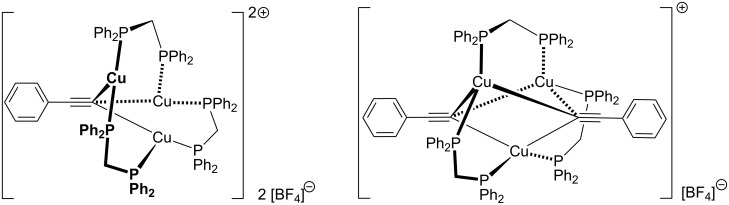
Schematic representation of the single crystal X-ray structures of copper(I) acetylide complexes [Cu_3_(μ_3_-C≡CPh)(μ-dppm)_3_][BF_4_]_2_ and [Cu_3_(μ_3_-C≡CPh)_2_(μ-dppm)_3_][BF_4_] [161].

In the first step, the alkyne π-coordinates to the copper(I) centre and can be easily deprotonated to give the acetylide complex (**B**), for example by sodium ascorbate [87], which is present in excess in the Sharpless–Fokin procedures [12]. A neighbouring copper(I) centre might also attract an acetylide ligand, which is shown in the equilibrium reaction on the right side of Scheme 18. These complexes [L_2_*_n_*_-1_Cu_2_]^+^–C≡CR^1^ (**B**) and [L_2_*_n_*_-1_Cu_2_]–(C≡CR^1^)_2_ (**B^inactive^**) might resemble the copper(I) acetylide complexes shown in Figure 4, whose structures have been determined by the group of García-Granda [161].

However, this equilibrium is unproductive, as only the coordination of an azide molecule to the neighbouring copper(I) ion (**C**) enables the formation of the copper(I) triazolide (**D**). It is noteworthy that in this mechanistic proposal, the σ-acetylide ligand binds to another copper(I) centre than the azide. Fokin and Finn have revised this picture in a more recent study [81], where they propose a bimetallic intermediate with binding of the azide and the σ-acetylide ligand at the same copper(I) centre (vide infra). A disputable and implausible intermediate (**C**→**D**) analogous to the highly strained metallacycle proposed by Sharpless (Scheme 13) [12] for the first C–N bond-forming step is included in a review article by van Maarseeven [18]. Fokin and Finn have omitted this structure in their original representation [160], but directly proceed from intermediate **C** to the product of ring contraction, viz*.* copper(I) triazolide complex **D**. The latter can be protonated to set free the triazole product and regenerate the active dinuclear catalyst.

A reaction calorimetry study by Finn gives no “clean” second order reaction profile in the CuAAC of benzyl azide (0.25 M) with phenylacetylene (0.45 M) in the presence of copper(II) sulfate (2 mM), sodium ascorbate (25 mM) and TBTA (4 mM) at 65 °C [80]. Instead, the thermogram suggests different catalyst species to be involved. In their subsequent mechanistic report [81], Fokin and Finn kinetically investigated the reaction of phenylacetylene (1 mM; 10–50 mM) with benzyl azide (1 mM; 10–50 mM) in a solvent mixture of dimethyl sulfoxide and aqueous buffer in the presence of copper(II) sulfate (0.05 mM; 0.020–0.25 mM) and TBTA (2:1 ratio ligand:metal) at room temperature and pH 8 by varying the concentrations of the different reagents while keeping all other parameters constant. Surprisingly, they found a first-order dependence of the rate law with respect to the concentration of copper ions. As with other ligands tested in this study, the reaction was zero order in the concentration of the azide. The finding that catalysis is inhibited by high concentrations of alkyne [160] was confirmed by determination of the alkyne’s rate order to be negative (−0.28 ± 0.2). However, in contrast to CuAAC reactions with other ligands, there was no “threshold” performance, i.e. kinetics was strictly continuous. Based on these findings, Donnelly suggested that the solid state complex [Cu_2_(μ-TBTA-κ^4^*N^2^*,*N^3^*,*N^3^’*,*N^3^’’*)_2_][BF_4_]_2_ is only a catalyst precursor (Scheme 8) [131]. In his opinion the catalytic species is likely to be mononuclear as the bridging coordination of one of TBTA’s triazole groups is probably very labile, so that the dinuclear complex can easily dissociate in solution. Donnelly saw this idea in concordance with the isolation of a mononuclear copper(I) triazolide as CuAAC intermediate [164]. Fokin and Finn, on the other hand, point out the “complex ways” [81], in which CuAAC catalysis responds to changes in concentration, type of ligand, presence of chloride ions, type of buffer and other parameters. In objection to Donnelly’s suggestion of a mononuclear pathway, they strongly emphasize the need for a second copper centre to assist the formation of the C–N bond between the azide and the acetylide, which is in accordance with evidence from structural studies [117,161,165–185] as well as quantumchemical calculations [125,186]. Fokin and Finn suggest that more than one mechanism might be operational under the conditions studied and stress that the first order rate dependence in the presence of TBTA is an exception, as a second order dependence was found for most other ligand systems with related structures (vide infra) [80–81]. Although many questions remain unanswered, the authors conclude that the main advantage of TBTA is to “keep the metal coordination chemistry ‘cleaner’ by providing a high local concentration of weakly binding arms, while at the same time allowing access to open coordination sites” [81].

Most tris(heteroarylmethyl)amine ligands (Table 1) [82] form copper catalysts with a “clean” second order rate profile with respect to the concentration of copper ions in reaction calorimetry experiments [80]. In a subsequent study, Fokin and Finn have examined the rate law for the CuAAC reaction in the presence of TBTA, (BimH)_3_, (BimC_4_A)_3_, (Bth)_3_ and (Py)_3_. For the first time, the authors have experimentally obtained a rate law for the reaction of benzyl azide with phenylacetylene in the presence of Tris buffer, in which all participants, i.e*.* alkyne, azide and copper complex, have an integer exponent (Equation 1) [81].

[1]
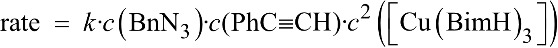


**Figure 5:**
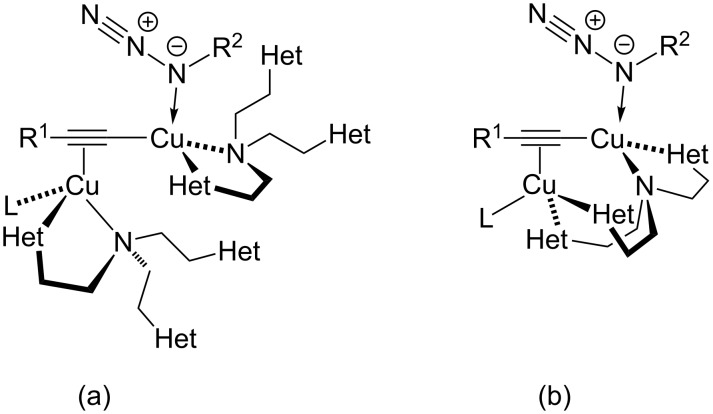
Acetylide-bridged dicopper complexes with tris(heteroarylmethyl)amine ligand(s) as key intermediates in the CuAAC reaction: (a) two tris(heteroarylmethyl)amine ligands bind to one copper centre each [81]; (b) one tris(heteroarylmethyl)amine ligand binds to both copper(I) centres [28,42] (L = halide, acetylide, hydroxide, triflate, or a neutral ligand, which would give a positive charge on this copper centre; Het = *N*-heterocyclic substituents such as benzimidazolyl, triazolyl, pyridyl or benzothiazolyl in the tripodal ligand; for examples please consult Figure 1, Table 1 and Figure 6).

Based on these findings, Fokin and Finn have suggested a pathway for CuAAC reactions in the presence of tris(heteroarylmethyl)amine ligands such as TBTA or (BimH)_3_, in which the azide binds to the same copper(I) centre as the σ-acetylide ligand. The second copper(I) ion, on the other hand, is only π-coordinated to the triple bond. A representation of the proposed acetylide-bridged dicopper species is shown in Figure 5. This is in contrast to the previously formulated general mechanistic picture (Scheme 18) [160], according to which the azide binds to another copper(I) centre than the σ-acetylide ligand.

Although a second-order rate dependence on the concentration of (BimH)_3_ had been experimentally determined [81], which hints at the involvement of two (BimH)_3_ ligands in the reactive species of the rate-determining step, structural proposal (a) from Fokin’s original mechanistic report [81] has lately been replaced by structure (b), which contains only one tris(heteroarylmethyl)amine ligand (Figure 5) [28,42].

**Scheme 19:**
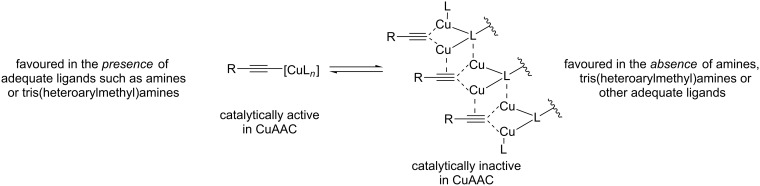
Off-cycle equilibrium between unreactive polymeric copper(I) acetylide species (right) and reactive monomeric copper(I) acetylides (left) as proposed by Fokin [42,63].

According to Fokin, the main advantage of using appropriate tris(heteroarylmethyl)amine ligands [82] such as TBTA in CuAAC catalysis is their “balanced” coordination chemistry with copper(I) ions: neither do these ligands bind too strongly, which would block the coordination sites on the copper(I) centres for the substrates, nor do they bind too weakly, which would permit the copper(I) acetylide species to form polymeric species [42,63] (Scheme 19). Fokin proposes that only well-defined non-aggregated copper(I) acetylides are reactive in CuAAC, whose concentration can be increased by adding ligands such as aliphatic amines or tris(heteroarylmethyl)amines, whereas polymeric copper(I) acetylide complexes are supposed to be unreactive.

It has also been shown that the presence of Tris buffer significantly decelerates the CuAAC reaction in the presence of (BimH)_3_ and even more so with TBTA over a standard range of concentrations [80]. However, in the absence of buffer, the copper complexes of (BimH)_3_ and (BimC_4_A)_3_ exhibited discontinuous kinetic behaviour: at low catalyst concentrations, the reaction stopped after a few turnovers, but at higher concentrations the usual high activity was observed, though it could only be marginally enhanced by further increasing the catalyst’s concentration. Similarly discontinuous behaviour was observed for the rate order with respect to the azide concentration. At low concentrations (10–20 mM) the observed rate order was 0.4, whereas the rate was independent of the azide’s concentration at higher concentrations (40–50 mM). When lowering the catalyst concentration, the rate was independent of the azide’s concentration throughout the given range of concentrations. This observation is attributed to the presence of chloride ions in the reaction mixture when Tris buffer is used, as the pH of the medium was identical for reactions in the presence and absence of Tris due to the buffer capacity of sodium ascorbate. A control experiment showed that addition of potassium chloride had an inhibitory effect on catalysis as well (approximately by a factor of 3), and that the discontinuous kinetics with regard to the catalyst’s and the azide’s concentration was not observed anymore. As only the rate order of the azide and not of the alkyne was influenced by addition of potassium chloride, it is suggested that chloride ions and the azide substrate have approximately the same affinity for copper(I) centres. Alkyne or acetylide ligands, on the other hand, coordinate much stronger to copper(I) ions and thus their rate order is not affected by the presence of chloride ions.

In another experiment, the concentration of copper ions was held constant and the concentration of ligand (BimH)_3_ was increased. It was observed that the initial amount of rapid catalytic activity before steady state was reached was higher with low ligand concentrations, i.e. the lower the ligand concentration, the faster the reaction at the beginning. In fact, the ligands listed in Table 1 can be sorted into two categories, namely group 1 that shows best catalytic activity at a metal/ligand ratio 1:1 [(BimH)_3_, (BimC_1_A)_3_, (BimH/S)_3_], and group 2 whose ligands perform optimally at a metal:ligand ratio of 1:2 [(BimC_1_H)_3_, (BimC_1_E)_3_, (BimC_1_E’)_3_, (BimC_4_A)_3_, (BimH/Me_2_)_3_]. However, even for group 1 ligands such as (BimH)_3_ the rate of reaction does not dramatically drop when excess quantities of the ligand are present. If only one species were present, we would expect the activity to give a peak at a 1:1 ratio and then drop, as excess ligands block coordination sites for the substrates and thus create a much less active copper species. This means that the observed marginal loss of activity in the presence of excess ligand is either due to a low equilibrium constant for the formation of the less active catalyst species or that at least two mechanistic pathways for catalysis exist. It is suspected that a “complex set of equilibria” is involved in the speciation of catalytically active copper(I) complexes and that the formation of different multinuclear copper aggregates is decisive [80–81].

**Figure 6:**
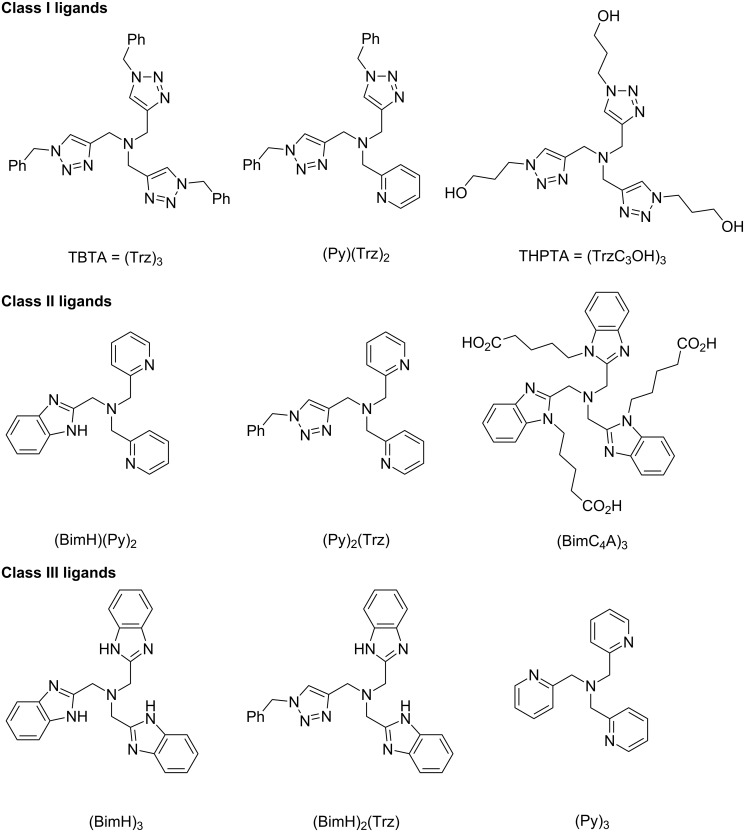
Categories of tris(heteroarylmethyl)amine ligands regarding their binding ability to copper(I) ions [83].

In 2010, Finn et al. published a comprehensive study comparing different (hybrid) tris(heteroarylmethyl)amine ligands [82] regarding their binding abilities, kinetic rate orders and catalytic performance under various conditions by calorimetry measurements [83]. The affinity of the heterocyclic arms to the copper(I) ions increases in the order triazole << pyridine < benzimidazole [81]. The different (hybrid) ligands are assigned to three different classes as shown in Figure 6.

- *Class I* ligands do not contain benzimidazole groups and therefore coordinate weakly to copper(I) ions. In aqueous media (H_2_O/DMSO 9:1), these ligands maintain their high catalytic activity even if the ligand is present in great excess (ligand/metal ratios of 2:1 and 4:1). In contrast, the catalytic performance with these ligands is bad in DMSO. TBTA is an example for this class.
- *Class II* ligands contain two or more benzimidazole or pyridine groups and coordinate strongly to copper. The catalytic activity of the corresponding copper complexes is retained in DMSO. In aqueous solutions, however, optimal catalytic performance is observed at ligand/metal ratios of 0.5:1 or 1:1. Beyond this maximum, the catalytic activity decreases dramatically as the ligand is added in excess. An example for class II is (BimH)(Py)_2_, an extremely active ligand in a 4:1 solvent mixture of DMSO and water at a ligand/metal ratio between 0.5:1 and 1:1. The rate order for the corresponding complex [Cu(BimH)(Py)_2_] was experimentally determined to be three in a 4:1 solvent mixture DMSO/H_2_O and a first order rate law was found in an aqueous medium (DMSO/H_2_O 1:9). The rate order was the same for differrent metal/ligand ratios.
- *Class III* contains ligands that form relatively inactive copper complexes over all metal/ligand ratios tested, for example (BimH)_3_ or (Py)_3_.

These observations can be explained by considering the relative donor strengths of solvent molecules and ligands.

In strong donor solvents such as DMSO, DMF and NMP, the solvent molecules compete with the substrates and the *N*-donor ligands for binding sites at the metal centre. Thus, weak ligands categorized in class I are replaced by solvent molecules and ligand-accelerated catalysis is diminished. Class II ligands, on the other hand, coordinate much more strongly to copper(I) ions than solvent molecules, even if strong donor solvents such as DMSO are used. Reactions thus proceed well in both strongly and weakly coordinating solvents as long as the ligands are not present in excess. As soon as the ligand/metal ratio exceeds a certain threshold (0.5 or 1), the catalytic activity drops dramatically as very stable complexes are formed which do not have free coordination sites for binding the azide substrate. Class I ligands are not able to form such stable inhibitory complexes and can thus be used in great excess in weakly coordinating solvents like water.

**Scheme 20:**
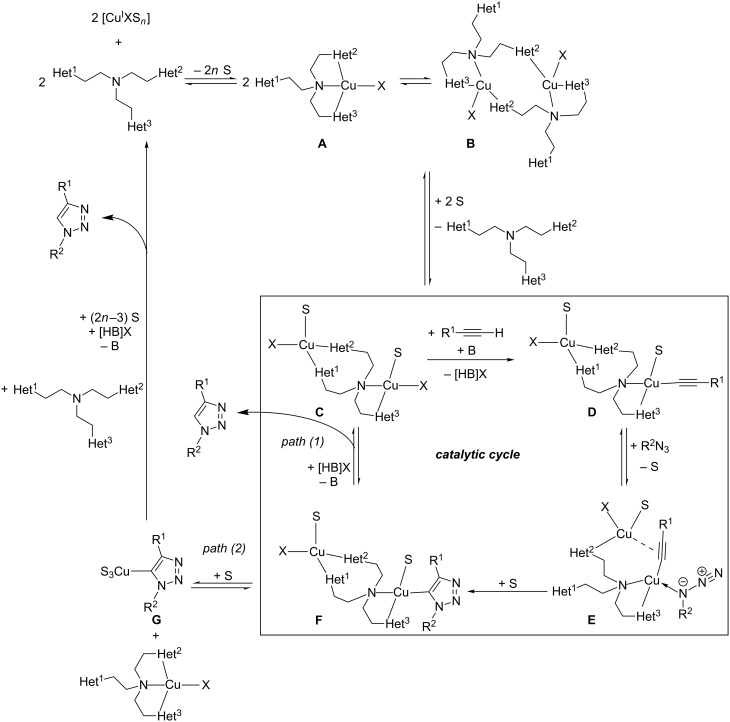
Mechanistic scheme for ligand-accelerated catalysis with tripodal tris(heteroarylmethyl)amine ligands in consideration of solvent coordination and formation of inhibitory species (S = solvent molecule; B = base; X = halide; *n* = integer number; Het^1^, Het^2^, Het^3^ = coordinating heteroaryl substituent such as benzimidazolyl, triazolyl, pyridyl or benzothiazolyl in the tripodal ligand; for examples please consult Figure 1, Table 1 and Figure 6) [83].

To summarize their mechanistic picture for CuAAC reactions in the presence of tripodal tris(heteroarylmethyl)amine ligands also taking into account the coordinating solvent molecules and inhibitory species, Finn et al. proposed the mechanistic picture shown in Scheme 20 [83].

Complexes **A** and **B** shown in the upper part of Scheme 20 are catalytically inactive: mononuclear complex **A** lacks a second copper centre to facilitate the C–N bond-forming step [125,186], and in complex **B** both copper(I) ions are coordinatively saturated so that the azide substrate cannot be bound. These inhibitory complexes **A**/**B** are formed when strong ligands (class II) are used in equimolar or excess amounts in solvents which can only coordinate weakly to copper(I) ions. With only 0.5 molar equivalents of class II ligands or by dissociation of weakly binding class I ligands, the catalytically active complex **C** can be formed, whose solvent ligands can be easily replaced to give the acetylide complex **D** and then complex **E**, in which both substrates are assembled for the crucial C–N bond-forming step. The pathway by which the copper triazolide complex **F** breaks down determines the overall rate order of reaction with respect to the concentration of copper(I) ions and ligand. In weak donor solvents, the dinuclear scaffold remains intact by regeneration of complex **C** (path (1)). As no change in the number of copper(I) ions present in the active species occurs in the catalytic cycle, this pathway would lead to a first-order dependence on the concentration of the dinuclear copper catalyst: rate = *k* · *c*(Cu_2_[tris(heteroarylmethyl)amine]). However, in strong donor solvents, dinuclear complex **F** breaks up to give two mononuclear complexes **A** and **G** (path (2)). This pathway is in accordance with a second-order dependence on the concentration of copper(I) ions and a first-order dependence on the ligand’s concentration, as one ligand and two copper centres need to be reassembled for one turnover: rate = *k* · *c*^2^(Cu^I^) · *c*(tris(heteroarylmethyl)amine). All in all, the following conclusions can be drawn: for CuAAC reactions in weak donor solvents, e.g. under aqueous conditions, weaker ligands (class I) are favourable in order to minimize the formation of inhibitory chelate complexes such as **B** or other unreactive polymeric species (Scheme 19). Stronger ligands (class II) are necessary in strong donor reaction media in order to facilitate the formation of dinuclear copper complexes needed for the mechanistic steps shown in Scheme 22.

The first DFT study on the CuAAC’s mechanism was carried out in 2005 by Sharpless and Fokin for the model reaction of propyne with methyl azide [13]. Geometry optimizations were carried out on the theoretical level B3LYP/6-311G(d,p) with subsequent single point energy calculations with basis set 6-311+G(2d,2p). Solvation energies for an acetonitrile or water solvent environment were calculated with the COSMO model at the B3LYP/6-311G(d,p) level. All energies disclosed in this report are enthalpies to which solvation energies and zero-point energies were added. Albeit only the mechanistic pathway featuring mononuclear copper species shown in Scheme 13 was investigated, which is not in accordance with the kinetic studies described above, there are still some results worth mentioning. The calculations show that π-coordination of propyne to the copper(I) complex leads to a decrease in the p*K*_a_ value of 9.8 units, i.e. the alkyne is greatly acidified from p*K*_a_ (propyne) ≈ 25 to p*K*_a_ (copper-coordinated propyne) ≈ 15. Reaction of the copper–alkyne π-complex with the azide in a concerted cycloaddition was shown to be very unlikely due to the high activation enthalpy of 27.8 kcal mol^−1^ (= 116.4 kJ mol^−1^). Even the copper-free uncatalyzed concerted reaction has a lower enthalpy of activation (25.7 kcal mol^−1^ = 107.6 kJ mol^−1^). Instead, the alkyne ligand is deprotonated to give the σ-acetylide complex. Such complexes are stable in aqueous solutions, even at acidic pH values [117]. The σ-acetylide complex cannot undergo a concerted reaction with the azide either, as the activation barrier for this step is too high as well (23.7 kcal mol^−1^ = 99.2 kJ mol^−1^). It has thus been proven that copper-catalyzed 1,3-dipolar cycloaddition reactions proceed via a stepwise mechanism. Indeed, the azide can replace one of the solvent ligands (acetonitrile or water) coordinating to the copper(I) centre. The ligator atom is the nitrogen next to the carbon. Starting from this resting state, the formation of the six-membered, highly strained copper(III) metallacycle (Scheme 13) was shown to be endothermic by 8.2 kcal mol^−1^ (34.3 kJ mol^−1^) for L = S = acetonitrile and 12.6 kcal mol^−1^ (52.8 kJ mol^−1^) for L = S = water. The activation barrier for this elementary step is 14.9 kcal mol^−1^ (62.4 kJ mol^−1^) for L = S = acetonitrile and 18.7 kcal mol^−1^ (78.3 kJ mol^−1^) for L = S = water, and is thus significantly lower than the barrier calculated for the uncatalyzed Huisgen cycloaddition (25.7 kcal mol^−1^ = 107.6 kJ mol^−1^). Without any significant activation barrier, this highly strained intermediate undergoes ring contraction to give the copper triazolide, from which the triazole product is released upon protonation. In fact, if this reaction is carried out in D_2_O, deuterium is incorporated at the C5 position of the triazole. Although these calculations for the stepwise mechanism shown in Scheme 13 can account for the regioselectivity and the observed rate increase compared to the uncatalyzed concerted Huisgen cycloaddition, this proposal is not in accordance with the fact that the rate law is second order with respect to the concentration of copper(I) ions in solution.

**Scheme 21:**
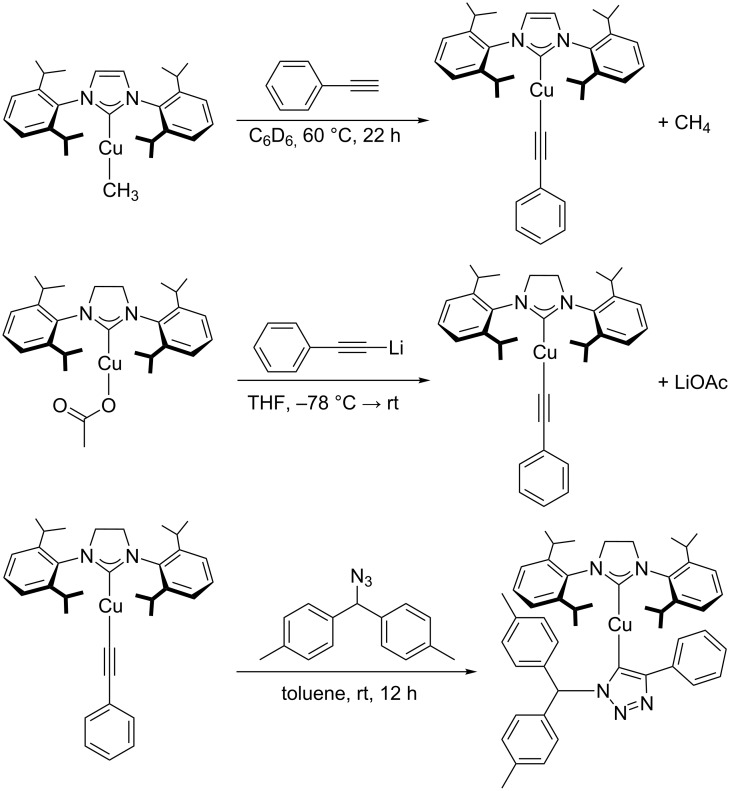
Synthesis of supposed intermediates in the CuAAC’s catalytic cycle [164,187].

The proposed intermediary formation of copper(I) acetylide as well as triazolide complexes has been experimentally supported by isolation and characterization of the NHC-copper(I) acetylide complexes [(IPr)Cu(C≡CPh)] [187] and [(SIPr)Cu(C≡CPh)] [164], as well as the NHC-triazolide complex [164] of the reaction with 4,4'-(azidomethylene)bis(methylbenzene) (Scheme 21).

Based on the isolation of these Click intermediates, it was suggested that the CuAAC can proceed via a mononuclear pathway when sterically encumbered copper(I) NHC-complexes are employed. This assumption has only recently been challenged (vide infra) [188].

Usually, however, copper(I) acetylides are polynuclear structures in which the acetylide ligands coordinate to two copper centres either in the unsymmetric σ,π-coordination mode (μ_2_-η^1^,η^2^) or in the symmetric σ,σ-coordination mode (μ_2_-η^1^,η^1^) [117,161,165–185]. It is consensus in all mechanistic investigations that π-complexes of the alkyne substrate with the copper(I) centres need to be formed in order to enable facile deprotonation to give the corresponding σ-complexes. The group of Mykhalichko has structurally investigated both copper(I) alkyne and acetylide complexes [117]. For the π-complexes, the authors found that the alkyne can either act as a bridging π-ligand for two copper centres (μ_2_-coordination mode), for example in the structure of [Cu_2_Cl_2_(HC≡CCH_2_OH)], or exclusively as a π-ligand for one copper(I) ion only, e.g*.* in [CuCl(HC≡CPh)]. According to the Chatt–Dewar–Duncanson model [189–191], the coordination of a copper(I) ion (3d^10^ 4s^0^) can be explained by a donating interaction L→M, in which electron density is transferred from the ligand to the metal centre, and a π-backdonation M→L from the metal to the ligand. In the case of a copper–alkyne π-complex, electron density is transferred from the bonding π-molecular orbital of acetylene to an unoccupied s- or p-orbital with σ-symmetry at the copper(I) centre (L→M donating interaction). On the other hand, a symmetrically suitable, fully occupied d-orbital of the copper ion can overlap with an antibonding π*-molecular orbital of the alkyne ligand (M→L back donation). As a consequence of populating the antibonding π*-MO of the alkyne, the C–C bond is elongated and the C–C–R geometry no longer linear, but bent. The transfer of electron density from the π-MO of the alkyne to the metal leads to positive partial charges on the alkyne’s carbon atoms and to an elongation of the C–C bond as well. Donation and back donation interactions are synergistic, i.e. an increase in one component leads to an increase in the other component. The interaction of copper(I) ions with acetylene has also been studied computationally [192–195]. Ab initio calculations by Frenking et al. have shown the metal-ligand coordinative bond in [Cu(HC≡CH)]^+^ to be stronger (*D_e_* = 40.6 kcal mol^−1^ = 170.0 kJ mol^−1^) than previously suspected [192–194]. The C–C bond length was calculated to be 1.242 Å and the bending of the acetylene moiety C–C–H was found to be 168.7° in this *C*_2_*_v_*-symmetric complex. In comparison, the bond length in free acetylene is 1.207 Å [196–197]. NBO analyses have shown that the metal–ligand interactions in [Cu(HC≡CH)]^+^ are mainly electrostatic and that the electron density distribution is T-shaped. This means that the donating interaction L→M greatly prevails, which is in accordance with an ESR study by the group of Watanabe [198].

**Figure 7:**
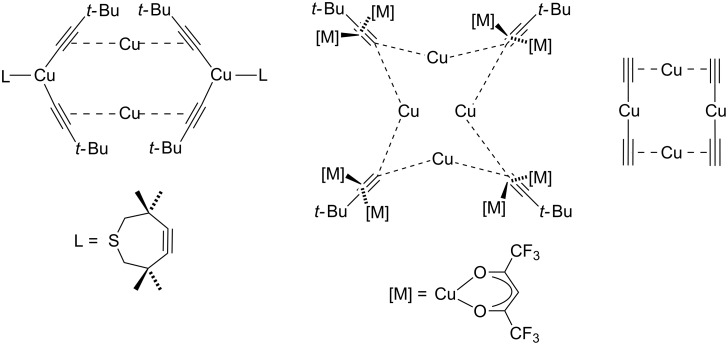
Tetranuclear copper acetylide complexes as reported by Weiss (left) [176] and Tasker (middle) [185] and model of tetranuclear copper(I) acetylides as resting state for mechanistic DFT studies [186].

The polarization of the C–H bond of terminal alkynes or acetylene in copper π-complexes substantially facilitates the deprotonation and formation of the corresponding acetylide complex [13]. The resulting Cu–C bond is so strong that the copper acetylide is even formed in strongly acidic media, for example in Cu_2_SO_4_ solutions with up to 25% H_2_SO_4_ [117]. The equilibrium between π-complex [Cu(HC≡CH)]^+^, monocopper acetylide [Cu(C≡CH)] and bisacetylide Cu_2_C_2_ is greatly influenced by the pH of the medium. The presence of halide ions and other ligands such as phosphines has a strong impact on speciation and nuclearity of these acetylide complexes, but they are in all cases complex polynuclear structures [117,161,165–185]. This is why our research group chose a tetranuclear model copper(I) acetylide and a dinuclear copper(I) acetylide complex with additional phenanthroline ligands as resting states for a DFT study on the CuAAC’s mechanism [186]. Figure 7 shows two tetranuclear copper(I) acetylide complexes described in literature, which justify the choice of a tetranuclear copper(I) acetylide model as resting state.

**Figure 8:**
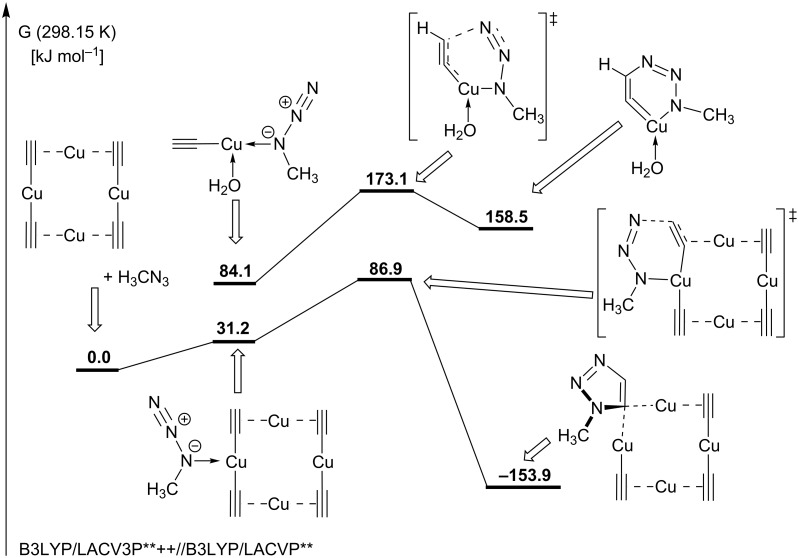
Gibbs free energy diagram for the computed mechanistic pathway of the CuAAC reaction starting from a tetranuclear copper(I) μ-acetylide model complex [186].

With these calculations, it was shown that the mononuclear pathway is greatly disfavoured due to the much higher Gibbs free energy of activation for the C–N bond forming elementary step: the barrier Δ*G*^≠^ for the mononuclear pathway was calculated to be 173.1 kJ mol^−1^, whereas the barrier for the putative pathway featuring tetranuclear complexes was only 86.9 kJ mol^−1^ (Figure 8). The high barrier of the mononuclear pathway is mainly due to the ring strain in the cyclic copper(III) intermediate: the sp-hybridized carbon atom in the fragment Cu=C=C prefers an angle of 180°, but this is impossible in a six-membered ring. The actual Cu=C=C angle was computed to be 131.4°. It is thus much more favourable for the alkenylidene carbon atom to bind to two copper(I) centres so that one Cu=C double bond is replaced by two Cu–C single bonds. However, the resulting six-membered copper(III) metallacycle is not stable and immediately undergoes reductive elimination to give the copper(I) triazolide complex.

**Figure 9:**
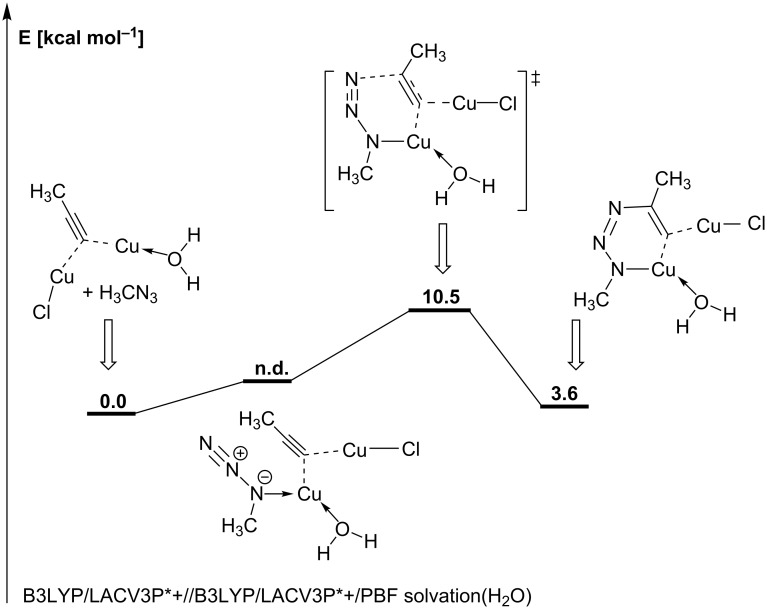
Energy diagram by Ahlquist and Fokin [125].

Ahlquist and Fokin published a similar DFT study on the theoretical level B3LYP/LACV3P*+ applying the PBF solvent model for water and supported the picture of dinuclear copper(I) complexes playing a vital role in the CuAAC reaction mechanism (Figure 9) [125]. All energies reported in their work are solution phase energies including zero point energy corrections. They found the overall energy barrier for a mononuclear pathway at 17 kcal mol^−1^ (= 71 kJ mol^−1^), which is in good agreement with the result published by Himo et al. in 2005 (18.7 kcal mol^−1^ = 78.3 kJ mol^−1^ for L = S = water) [13]. For the dinuclear pathway, they chose dicopper species with water and acetylide or chloride as spectator ligand. In the resting state, the acetylide is σ-coordinated to one copper(I) centre, to which an aqua ligand is coordinated as well. The second copper centre with an additional chloride or acetylide spectator ligand strongly interacts with the C1 atom of the acetylide, but less so with the C2 atom. Only in the presence of two identical spectator ligands was a strict μ_2_-η^1^,η^2^ binding mode observed. The calculated energy of activation for the addition of azide to the dicopper chloride complex was found to be 10.5 kcal mol^−1^ (= 43.9 kJ mol^−1^) and thus significantly lower than for the mononuclear pathway.

**Scheme 22:**
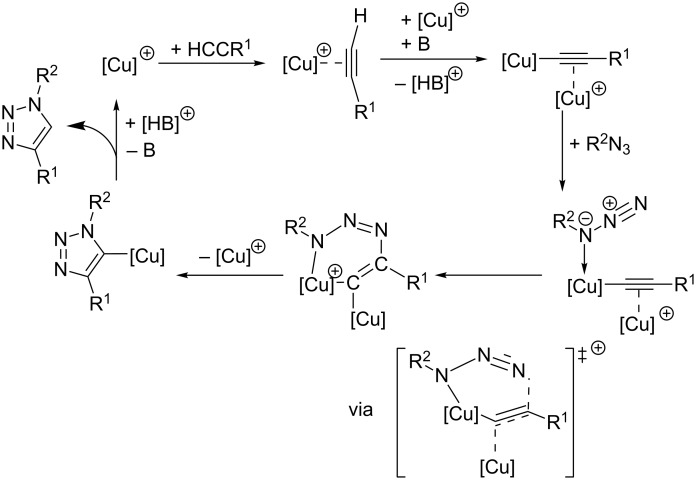
Mechanistic proposal for the CuAAC reaction based on DFT calculations by Fokin [125] and our group [186] ([Cu] stands for a copper(I) complex with the suitable number of ancillary ligands).

These energy diagrams (Figure 8 and Figure 9) translate to the general mechanistic proposal shown in Scheme 22.

This mechanistic picture was supported by recent calculations carried out by the group of Cantillo [199]. In their DFT study, the authors compare different mechanistic proposals from literature on the same level of theory (B3LYP/LANL2DZ, solvent model CPCM for water). In conclusion, they confirm dinuclear copper acetylides to be the essential catalytic intermediates, whereas the corresponding copper alkyne π-complexes need to be deprotonated before the crucial C–N bond forming step can take place. The computed Gibbs free energy barrier for the CuAAC reaction of methyl azide with propyne was found to be 16.0 kcal mol^−1^ (= 70.0 kJ mol^−1^) for the formation of the 1,4-disubstituted triazole, and 20.4 kcal mol^−1^ (= 85.4 kJ mol^−1^) for the reaction giving the 1,5-disubstituted regioisomer.

**Figure 10:**
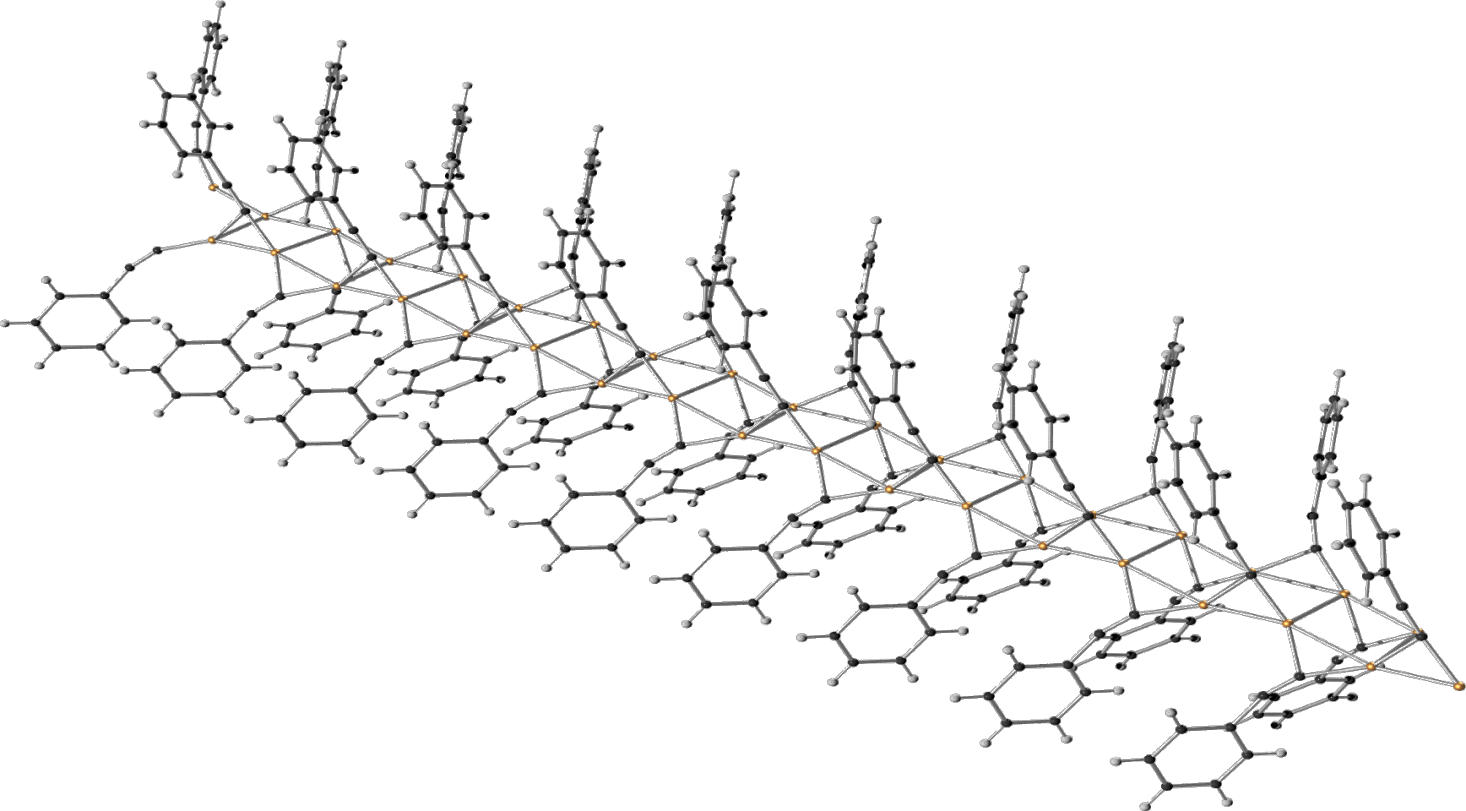
ORTEP plot [202–203] of the X-ray powder diffraction crystal structure of (phenylethynyl)copper(I) [(PhC≡CCu)_2_]*_n_* by Che et al. [184,204].

In 2010, the group of Heaney presented experimental evidence for the participation of dinuclear alkynylcopper(I) complexes in CuAAC reactions [87]. (Arylethynyl)- and (alkylethynyl)copper(I) compounds usually form polymeric aggregates [(RC≡CCu)*_n_*] of low solubility [200–201]. For example, X-ray powder diffraction studies have shown the insoluble yellow (phenylethynyl)copper(I) [(PhC≡CCu)*_n_*] to consist of an infinite Cu–Cu ladder structure (*n* = ∞) [184]. Both copper ions in this ladder polymer [(PhC≡CCu)_2_]*_n_* adopt the same μ-η^1^,η^2^-C≡C bridging mode (Figure 10).

**Scheme 23:**
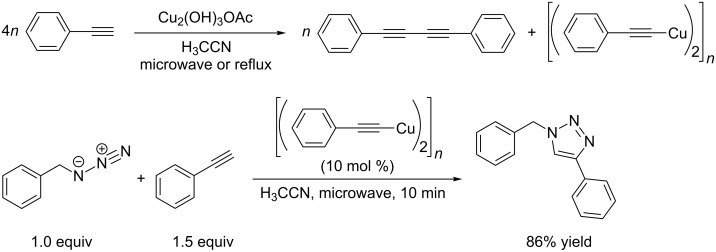
Synthesis of [(PhC≡CCu)_2_]*_n_* as co-product in the Glaser coupling of phenylacetylene in the presence of copper(II) hydroxyacetate in acetonitrile, and application of [(PhC≡CCu)_2_]*_n_* as catalyst in the CuAAC test reaction of phenylacetylene with benzyl azide [162].

The distance between the two copper(I) ions was found to be between 2.49 and 2.83 Å. The group of Heaney recognized that this intermetallic distance was in the same range as calculated by Ahlquist and Fokin for the transition state in CuAAC reactions featuring dinuclear model structures (2.54 Å and 2.64 Å for chloride/water and acetylide/water as spectator ligands, respectively) [125]. Heaney et al. concluded, that [(PhC≡CCu)_2_]*_n_* should thus be a perfect catalyst for CuAAC reactions, as the two copper ions needed for the crucial elementary steps are extremely well positioned for catalytic performance [162]. Experiments were carried out in acetonitrile at 100 °C with microwave irradiation in order to facilitate the heterogeneous reaction. With 10 mol % of catalyst [(PhC≡CCu)_2_]*_n_*, the reaction between phenylacetylene and benzyl azide reached 86% conversion within ten minutes (Scheme 23). The [(PhC≡CCu)_2_]*_n_* catalyst was recovered and re-used with similar results.

When 4-tolylacetylene was used as substrate in combination with [(PhC≡CCu)_2_]*_n_* as catalyst, ligand exchange at copper(I) took place and the product mixture contained 10% 1-benzyl-4-phenyl-1*H*-1,2,3-triazole and 85% 1-benzyl-4-(4-tolyl)-1*H*-1,2,3-triazole. The recovered yellow solid was used in the reaction of 4-tolylacetylene with benzyl azide thereafter and this reaction yielded 85% 1-benzyl-4-(4-tolyl)-1*H*-1,2,3-triazole. When no alkyne substrate was added, the azide reacted with a stoichiometric amount of [(PhC≡CCu)_2_]*_n_* to give 1-benzyl-4-phenyl-1*H*-1,2,3-triazole in 85% yield, as well as a brown insoluble residue, which was regenerated to [(PhC≡CCu)_2_]*_n_* by addition of phenylacetylene. It was also shown that under the given reaction conditions, the source of protons was the alkyne, as the reaction with 1-[D]-2-phenylacetylene proceeded with quantitative incorporation of deuterium at the triazole’s C5 position. In a subsequent study, the group of Heaney carried out CuAAC reactions under the original Sharpless–Fokin conditions [12]. Using 1 mol % copper(II) sulfate in a solvent mixture of *tert*-butanol/water in the presence of 10 mol % sodium ascorbate for the CuAAC test reaction of benzyl azide with phenylacetylene, the authors observed a transient yellow colour of the reaction mixture. With larger amounts of copper(II) sulfate, the known alkynylcopper(I) ladderane complexes [184] could be isolated and identified [87].

**Scheme 24:**
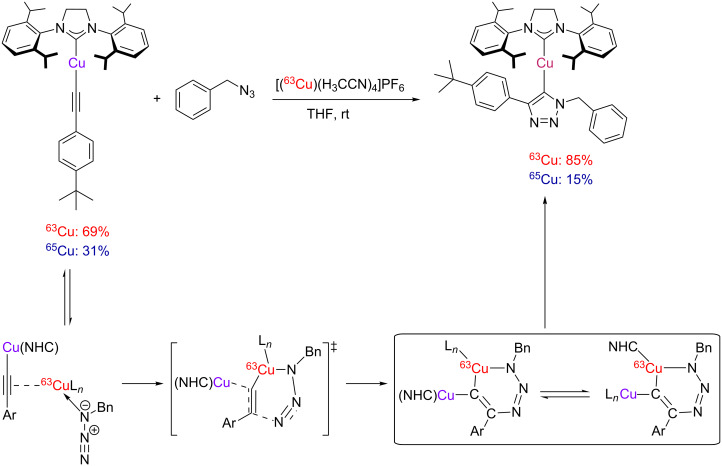
Mechanistic explanation for the isotopic enrichment in the product triazolide in the presence of the isotopically pure exogenous catalyst [(^63^Cu)(H_3_CCN)_4_]PF_6_ (with Ar = 4-*t*-BuPh, NHC = SIPr) [188].

Very recently, Fokin has presented direct evidence for the participation of a dinuclear copper intermediate in the CuAAC reaction mechanism [188]. In the first part of this study, the authors prepared the mononuclear σ-bound acetylide complex (SIPr)Cu(acetylide) [164]. With the help of real-time heat flow calorimetry, the authors monitored the progress of the reaction between this copper(I) acetylide complex and benzyl azide. The global heat output graph showed that there was no detectable conversion at all after one hour at 35 °C with tetrahydrofuran as solvent. However, when 5 mol % of [Cu(PPh_3_)_2_]NO_3_ were added, the CuAAC reaction was completed within 20 minutes under otherwise identical conditions. Similar results were obtained with [Cu(PPh_3_)_3_]Br, [Cu(PPh_3_)_3_]NO_3_, CuI/TTTA and CuI/triethylamine as soluble copper(I) species, and in chloroform and dimethylformamide as alternative solvents. These experiments show that monomeric copper(I) acetylide complexes are unreactive towards organic azides. In contrast, our group had observed the reaction of a similar complex (SIPr)Cu(acetylide) with the sterically encumbered 4,4’-(azidomethylene)bis(methylbenzene) in our study on the isolation of a copper(I) triazolide complex without the need for an additional source of copper(I) ions. Even though we had inferred that “dinuclear copper complexes are not mandatory for Huisgen–Sharpless Click reactions, since even mononuclear copper(I) acetylides with extreme steric shielding react with bulky organoazides at room temperature”, we had at the same time voiced our concern that “NHC dissociation, acetylide ligand exchange and formation of dinuclear complexes” still need to be ruled out in order to verify this conclusion [164]. By means of crossover experiments with isotopically enriched [(^63^Cu)(H_3_CCN)_4_]PF_6_ (1 equivalent) and the aforementioned (SIPr)Cu(acetylide) complex with natural isotopic distribution, Fokin and co-workers have now indeed shown that dinuclear copper complexes are essential for the CuAAC reaction to take place. With benzyl azide as substrate, an isotopic enrichment of approximately 50% was observed, namely from the naturally occurring ratio of 69(^63^Cu):31(^65^Cu) in the substrate complex (SIPr)Cu(acetylide) to 85(^63^Cu):15(^65^Cu) in the triazolide product complex (SIPr)Cu(triazolide). In order to find out at which stage isotopic scrambling of ^63^Cu and ^65^Cu occurs, mixtures of [(^63^Cu)(H_3_CCN)_4_]PF_6_ (1 equivalent) with the starting material acetylide (SIPr)Cu(acetylide) and with the triazolide product complex (SIPr)Cu(triazolide) were prepared, but with neither of them was any isotope exchange detected. Only in the presence of both the azide *and* the alkyne was isotopic scrambling between the NHC-copper complex and [(^63^Cu)(H_3_CCN)_4_]PF_6_ observed. It was thus suggested that two equivalent copper(I) centres participate in the rate-determining C–N bond-forming step of the CuAAC reaction mechanism and that the ligands on these copper(I) ions can exchange rapidly after the first C–N bond has been formed. This unusual dissociation of the copper–NHC bond can be explained by assuming a formal oxidation state of +III at the copper centre. Because of the low electron density at the copper(III) centre, the Cu→C(carbene) backbonding interaction is supposed to be substantially weakened, which allows for a “rapid exchange of the NHC ligand between the two copper atoms” in the intermediate shown in Scheme 24 [188].

Fokin thus draws the conclusion that these results do not only support the mechanistic picture given in Scheme 22, but also confirm that the CuAAC reaction can only take place when (at least) two copper(I) centres cooperate in the crucial mechanistic steps. It remains an unresolved question whether the formation of the triazolide species with 48% yield after twelve hours (Scheme 21) [164] can be attributed to a very slow mechanistic pathway with mononuclear species or to catalysis with traces of a reactive dinuclear copper(I) species in analogy to Fokin’s suggestion.

**Scheme 25:**
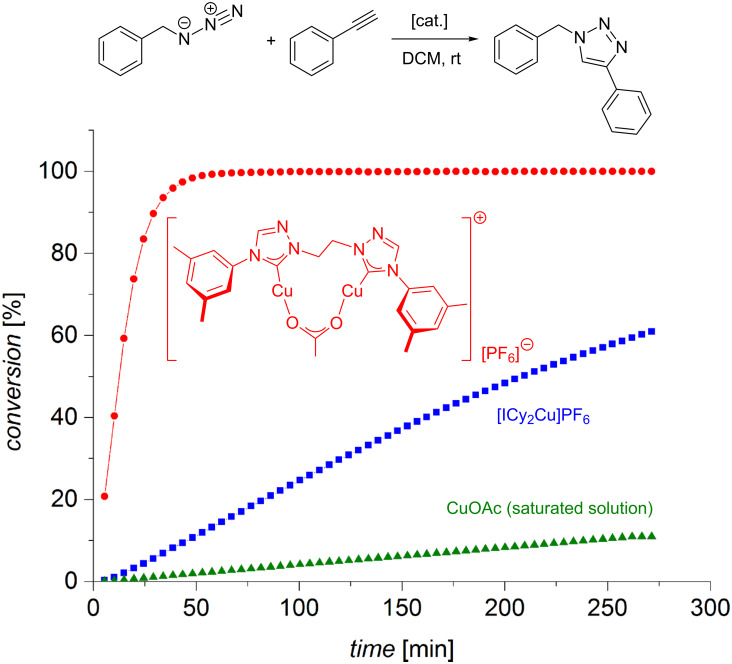
Homogeneous CuAAC catalysis with a bistriazolylidene dicopper complex (0.5 mol %) and comparison with [ICy_2_Cu]PF_6_ (1.0 mol %) and CuOAc (saturated solution) [163].

Based on the assumption that the interplay of two copper(I) ions is essential for the CuAAC reaction to take place via the mechanistic pathway shown in Scheme 22, our group has recently presented the first molecularly defined dinuclear catalyst system for homogeneous CuAAC reactions in organic solvents [163]. We suppose that the labile acetate ligand dissociates from the precatalyst so that the acetylide and the azide can be bound to the two copper(I) centres in order to facilitate the cycloaddition reaction (Scheme 25).

Due to the highly modular synthesis of this dicopper acetate complex, the characteristics of this catalyst can be tuned according to specific requirements of the substrates. Moreover, we envisage substantial new insights from kinetic studies with this molecularly defined catalyst system.

## Conclusion

Most mechanistic studies on the CuAAC reaction have been carried out by using mixtures of copper(I) precursors and additives. However, the structure of the catalytically active species with these “black box” reagent mixtures is unknown and structural identity and concentration of the active copper(I) species might even change [42] in the course of CuAAC catalysis. Thus, kinetic experiments can only provide limited mechanistic insights. Nevertheless, it appears unquestionable that copper(I) acetylide species tend to form aggregates and that CuAAC catalysis profits immensely from the interplay of at least two copper(I) centres. DFT studies strongly support this hypothesis [125,186,199]. In the light of Fokin’s direct evidence for the participation of a dinuclear copper intermediate [188], it remains unclear whether mononuclear copper complexes such as those formed from [(NHC)_2_Cu]PF_6_ are the actual catalytically active species or whether these precatalysts need to form aggregates to enable the interplay of at least two copper centres. Future research efforts in this field may tackle the following challenges: Can we isolate and structurally characterize dicopper acetylide intermediates of CuAAC reactions? Is the C–N bond formation always the rate-determining elementary step? Can the p*K*_a_ value of the coordinated alkyne substrate be determined, and what influence does the addition of acids and bases to the reaction mixture have on the CuAAC’s mechanism? Will the rate-determining step change depending on the acidity of the medium? How does dioxygen interfere with the dinuclear complexes needed for efficient CuAAC catalysis? Are dinuclear CuAAC catalysts decomposed by dioxygen by an analogous mechanistic pathway as suggested for oxidation reactions mediated by multi-copper enzymes such as tyrosinase or catechol oxidase [205–209]?

In view of the remaining questions regarding the CuAAC’s reaction mechanism and the specific structure of the catalytically active species, Fokin has lately pointed out that “superb catalysts are waiting to be found if we are adventurous enough to accept the uncertainty of not knowing the precise structure of the active catalytic species” [42] and Meldal speculated that “the detailed structural secrets of the transition state responsible for the extreme rate enhancement and selectivity in the Cu(I) catalyzed triazole formation will not be unambiguously determined in near future” [19]. Since then, many more insights into the mechanism of CuAAC have been gained, and we enthusiastically anticipate subsequent studies with molecularly defined copper(I) catalysts to unveil the remaining secrets of the CuAAC reaction’s mechanism.
